# Big data and deep learning for RNA biology

**DOI:** 10.1038/s12276-024-01243-w

**Published:** 2024-06-14

**Authors:** Hyeonseo Hwang, Hyeonseong Jeon, Nagyeong Yeo, Daehyun Baek

**Affiliations:** 1 School of Biological Sciences, Seoul National University, Seoul, Republic of Korea; 2 Interdisciplinary Program in Bioinformatics, Seoul National University, Seoul, Republic of Korea; 3 Genome4me Inc., Seoul, Republic of Korea

**Keywords:** Machine learning, Functional genomics, Computational models, Genetic databases, Transcriptomics

## Abstract

The exponential growth of big data in RNA biology (RB) has led to the development of deep learning (DL) models that have driven crucial discoveries. As constantly evidenced by DL studies in other fields, the successful implementation of DL in RB depends heavily on the effective utilization of large-scale datasets from public databases. In achieving this goal, data encoding methods, learning algorithms, and techniques that align well with biological domain knowledge have played pivotal roles. In this review, we provide guiding principles for applying these DL concepts to various problems in RB by demonstrating successful examples and associated methodologies. We also discuss the remaining challenges in developing DL models for RB and suggest strategies to overcome these challenges. Overall, this review aims to illuminate the compelling potential of DL for RB and ways to apply this powerful technology to investigate the intriguing biology of RNA more effectively.

## Exploring the complex world of RNA with deep learning

This review spotlights the revolutionary role of deep learning (DL) in expanding the understanding of RNA. RNA is a fundamental biomolecule that shapes and regulates diverse phenotypes including human diseases. Understanding the principles governing the functions of RNA is a key objective of current biology. Recently, big data produced via high-throughput experiments have been utilized to develop DL models aimed at analyzing and predicting RNA-related biological processes. This review emphasizes the role of public databases in providing these big data for training DL models. The authors introduce core DL concepts necessary for training models from the biological data. By extensively examining DL studies in various fields of RNA biology, the authors suggest how to better leverage DL for revealing novel biological knowledge and demonstrate the potential of DL in deciphering the complex biology of RNA.

This summary was initially drafted using artificial intelligence, then revised and fact-checked by the author.

## Leveraging big data with deep learning in RNA biology

Over the last decade, deep learning (DL) has proven to be a versatile tool in biology, aiding in multiple breakthroughs in structural biology, genomics, and transcriptomics^1^. The power of DL lies in its unique ability to harness the potential of big data^2^. Recently, big data have been rapidly accumulating in multiple domains of biology^3^. In particular, high-throughput experiments based on RNA sequencing (RNA-seq) have led to the generation of massive amounts of RNA biology (RB) data^4^. Analyzing these big biological data with DL has led to novel scientific discoveries about RNA and related biological processes. Therefore, it would be beneficial to review the current progress of DL in RB, focusing on the role of big data.

DL models are multilayered artificial neural networks that learn to generate representations of input data. These models can perform downstream tasks such as regression, classification, and generation. They have higher degrees of freedom than do conventional machine learning algorithms and thus can effectively learn representations from high-dimensional data^5^. This property has allowed DL models to achieve groundbreaking success in various fields, including computer vision^6^, natural language processing^7^, and structural biology^8^. Constructing such effective DL models requires sufficiently large datasets. However, the availability of such datasets is often a major bottleneck. Auspiciously, the amount of biological data has exploded due to the universal use of high-throughput experiments in RB.

RB is an integrative field of biology in which biological processes involving diverse types of RNA are studied. The utilization of DL in this field has been driven by high-throughput experiments using RNA-seq. These experiments have led to the generation of large-scale biological data for systematically examining RNA-related phenomena. Among the representative examples are cross-linking and immunoprecipitation (CLIP) for protein binding analysis^9,10^, N^6^-methyladenosine-sequencing (m6A-seq) for RNA modification analysis^11^, DMS-seq for RNA structure analysis^12^, and single-cell RNA sequencing^13^.

Although DL is a promising approach for revealing the mechanisms underlying RNA-related biological processes, this approach is not without challenges. First, most of the popular DL architectures and algorithms have not been optimized for biological data and tasks. Second, obtaining a training dataset of sufficient size and quality is often difficult. Third, the difficulty in understanding the prediction of DL models often impedes the conception of scientific hypotheses, which requires understanding the causal relationship between the input and the output^14^. Nevertheless, successful examples of employing DL in various domains of RB demonstrate the feasibility of acquiring biological insights from big biological data. This goal can be achieved by selecting and optimizing adequate strategies and techniques of DL regarding the characteristics of transcriptomic data.

This review provides an introductory guide to employing DL for novel discoveries in RB. First, we review widely used large public databases for RB, focusing on their utility in building DL datasets. Next, we describe how popular DL methods can be employed to exploit and complement the characteristics of RB datasets. We then introduce the methods for encoding various types of RB data into input features and popular deep neural network architectures suitable for processing these features. The primary goal of these sections is to provide a foundational understanding for designing and training DL models that can learn robust representations of big RB data. Subsequently, we review successful applications of DL in various domains of RB. Finally, we discuss the desiderata and open challenges in applying DL to RB.

## Public sources of large-scale RNA biology data

Training a DL model for RB applications starts with obtaining a suitable training dataset. Multiple de facto standard datasets for training and benchmarking exist for conventional DL applications, such as computer vision and natural language processing. However, such datasets are scarce in RB. Therefore, researchers often have to construct new training datasets by collecting data from existing public databases. When building training datasets from public biological databases, filtering, labeling, and normalizing the experimental data using metadata are essential. Metadata are collections of sample or experiment-associated information, often including experiment type, experimental group, sample type, organism, health status, and sequencing platform metadata^15,16^. Most public biological databases provide metadata, but their format, stringency, and completeness vary widely. In this section, we review such public databases for RB, focusing on experimental data types and metadata (Table 1).

**Table 1:** Public databases for large-scale RB data.

Name	RNA biology data type	Format	Datapoints	Biosample source	Access	Metadata	QC policy	Ref.
GEO	Microarray, RT‒PCR, NGS, and SAGE	Diverse formats, including tables (TXT/CSV/TSV), WIG, bigWig, bedGraph, GFF, GTF, and native microarray files (e.g., CEL)	6,970,856 total samples with 1,865,802 non-SRA RNA samples	Cell lines, tissues, and others	Open	Metadata fields include organism, cell line, genotype, treatment, time, molecule, single- or paired-end, and instrument model.	It is recommended that submitters follow MIAME or MINSEQE standards. Only basic checks for obvious errors are performed by curators upon submission.	^17^
SRA	NGS, including RNA-seq and its variants, such as ncRNA-seq, miRNA-seq, and bisulfite-seq	FASTQ, BAM, CRAM, SFF, and HDF5	10,923,374 public RNA experiments	Cell lines, tissues, and others	Partially restricted	Metadata fields include assay type, source, depth, disease, platform, sex, and treatment.	Automated and manual checks to ensure data integrity and quality.	^296^
ArrayExpress	46 types of functional genomics experiments based on microarray and NGS	Native microarray files (e.g., CEL), tab-delimited TXT, FASTQ, and BAM	77,794 total studies with 13,988 RNA-seq studies and 51,250 array studies	Cell lines, tissues, and others	Open	Metadata fields include description, protocols, sample annotation, author information, and sequencing library specification.	It is recommended that submitters follow MIAME or MINSEQE standards. Manual curation, including annotation refinement, is performed by trained bioinformaticians upon submission.	^27^
ENA	NGS, including RNA-seq and its variants, such as ncRNA-seq, miRNA-seq, and bisulfite-seq	FASTQ, BAM, CRAM, SFF, and HDF5	28.9 M total runs	Cell lines, tissues, and others	Partially restricted	Metadata fields include taxon, instrument, platform, nominal length, library, read count, base count, and author. Sample type-specific community-developed reporting standards and INSDC missing value reporting terms can be used.	Curators perform sorting, error correction, and taxonomy assignment.	^28^
ENCODE	Functional genomics experiments, including RNA-seq, CLIP, and microarray	FASTQ, BAM, BIGWIG, and BED	23,521 total experiments with 4747 RNA experiments	Cell lines, tissues, and others	Open	Metadata fields include assay, target, organism, biosample, organ, cell, system, sex, disease, life stage, treatment, platform, status, audit, pipeline, and assembly.	Audits and QCs performed by the ENCODE DCC. Experiments are flagged for imperfections, including low read depth, missing replicates, missing spike-in, and poor library complexity. Unified analysis pipelines are applied to some experiments.	^15^
TCGA	RNA-seq	Diverse formats, including XML, TXT, CEL, BAM, VCF, and MAF	25,018 RNA-seq data	Human tissues	Partially restricted	Metadata fields include patient information, sample, portion, slide, analyte, tumor type, timestamp, and creator.	Data are generated according to SOPs, which are available from NCI’s Biospecimen Research Database.	^35^
ICGC	RNA-seq, including miRNA-seq	VCF and BAM	21,215 files from 10,177 donors	Human tissues	Partially restricted	Metadata fields include type, submitter, donor, specimen, date, coverage, number of genes, platform, strandedness, and read length.	Expected sequencing coverage, somatic variant validation (differs in matched normal).	^297^
GTEx	RNA-seq	GCT, GTF, BAM, CRAM, VCF, and tables	17,382 RNA-seq samples and 88 ONT DRS samples	Human tissues	Partially restricted	Metadata fields include sample ID, technology, comments of the pathologist, RNA integrity number, tissue type, interval between death and tissue stabilization, time spent in fixative, and alignment metadata.	Data are generated according to their own pipeline and methods.	^279^
FANTOM	CAGE and its variants (DeepCAGE, nanocage, CAGEscan, HeliScopeCage), RNA-seq and its variants (small RNA-seq, directional RNA-seq), and ChIRP-seq	FASTQ, BED, and BAM	3940 samples (as of FANTOM5)	Mammalian cell lines and tissues	Open	Metadata fields include source, category, species, sex, age, provider, cell type, tissue, and protocol.	Data are generated according to their own pipeline and methods.	^30^
K-BDS	RNA-seq, scRNA-seq, and microarray	FASTQ, FASTA, BAM, SAM, VCF, and TSV	4505 RNA-seq (including scRNA-seq) experiments and 497 RNA microarray data	Various species including human, mouse, and microorganisms. Metagenomic data are included	Open	Mandatory metadata fields for human NGS data include sample name, organism, tissue, biomaterial provider, isolate, and sex. Optional fields include type, cell line, cell type, cell subtype, culture collection, biological replicate, treatment, karyotype, age, development stage, disease, disease stage, health state, phenotype, population, race, and ethnicity.	Automatic data inspection followed by manual examination and curation by data experts to enable data reanalysis.	^298,299^
HCA	scRNA-seq	BED, BAM, HDF5, FASTA, and FASTQ	52.5 M cells from 7.9 k donors	Healthy human and mouse tissues	Open	Metadata fields include cell line, donor organism, specimen, analyses protocol, and experiment protocol	scRNA-seq data are submitted in unprocessed formats, and HCA processes the data using standardized processing pipelines to produce alignment, quantification, and QC metrics.	^224^

### GEO and SRA

The Gene Expression Omnibus (GEO) is a public repository of gene expression data, including RNA-seq and microarray data, managed by the NCBI^17^. As of January 2024, GEO contains expression data for more than 6.97 million biosamples, including more than 1.86 million RNA samples. One representative example of public data archived in the database is the National Institutes of Health (NIH) Roadmap Epigenomics Project, which provides 111 reference human epigenomes with gene expression profiles^18^. Since GEO does not store raw data from high-throughput sequencing experiments, the data are archived in the Sequence Read Archive (SRA). SRA was established to provide a public archive of high-throughput sequencing data in conjunction with GEO^19^. As of January 2024, SRA has hosted more than 10.9 million publicly available RNA experiments.

While GEO serves as a unique and irreplaceable data source for RB researchers, one of its limitations is its lenient control over metadata items and terms, resulting in the incoherence and fragmentation of metadata^20,21^. Aliases and missing metadata fields often hamper the automated filtering and labeling of experimental data. While there have been several efforts to mitigate this issue^21,22^, improving the integrity of GEO metadata remains a challenge. Moreover, ensuring the quality of the data and analysis pipeline is primarily the responsibility of the submitters, introducing a potential source of data inconsistencies and imperfections when compiling a training dataset from GEO. Therefore, while GEO is an unparalleled source of RB data, cautious filtering, validation, and normalization are required to assemble a training dataset from the database.

### ENCODE

The Encyclopedia of DNA Elements (ENCODE) project, driven by the ENCODE consortium, which is organized and funded by the National Human Genome Research Institute (NHGRI), provides a wide range of functional genomics data^23^. In the third phase of ENCODE (ENCODE 3), the quality and quantity of public functional genomics were improved by adding 5992 experiments, including 170 eCLIP experiments, 78 RBNS experiments, 155 RAMPAGE experiments, 198 miRNA/small RNA-seq experiments, and 340 total/poly(A) RNA-seq experiments^24^. Moreover, ENCODE contains tissue expression data (EN-TEx), which include gene expression profiles and 15 functional genomics assay data from 30 human tissues^25^. One of the most prominent features of the database is that it enforces standardized experiment-specific quality control methods for both the data and the metadata^26^. The experimental data are audited, and audit flags are placed on a variety of potential imperfections in the data. Moreover, the use of controlled fields and terminologies for each metadata entry is enforced. ENCODE also provides unified analysis pipelines for multiple types of RNA experiments, improving the reproducibility of the analyses. Another notable feature of ENCODE is that it supports a powerful filtering functionality, allowing researchers to select data based on the assay type, target gene, target organism, cell line, sequencing platform, and many other features. Therefore, ENCODE is an essential source of quality-controlled data for constructing training datasets for functional genomics DL models.

### ArrayExpress & ENA

ArrayExpress is a public archive for functional genomic data managed by the European Bioinformatics Institute (EBI)^27^. This archive includes 46 types of functional genomics data, including 13,988 RNA-seq studies and 51,250 array studies. The submitted data are enforced to meet minimal metadata requirements and are manually curated by bioinformaticians, but the database does not provide detailed audit information. The raw data from high-throughput sequencing experiments in ArrayExpress are archived in the European Nucleotide Archive (ENA). The submitted data are required to meet quality standards. The ENA manages the metadata to ensure that the minimal standards are met using controlled fields and vocabulary^28^.

### FANTOM

The Functional Annotation of the Mammalian Genome (FANTOM) consortium aims to improve the understanding of the gene regulation network^29^. The FANTOM database provides atlases of gene regulatory elements and noncoding RNAs^30,31^. The database primarily hosts RNA-seq and cap analysis of gene expression (CAGE) data. CAGE accurately maps transcription start sites (TSSs) by pulling down 5’ caps^32^. The current phase of the project, FANTOM6, is focused on characterizing the global regulatory effect of human lncRNAs^33^.

### GTEx, TCGA, and ICGC

Genotype–Tissue Expression (GTEx) is a project initiated by the NIH to provide RNA-seq data for various human tissues associated with genomic sequences and map tissue-specific and global expression quantitative trait loci (eQTL)^34^. The completed GTEx v8 project encompasses 54 tissue types of human adults, and the developmental GTEx (dGTEx) project, which is ongoing, includes tissue samples from infants and juveniles. The Cancer Genome Atlas (TCGA), managed by the National Cancer Institute (NCI), also provides RNA-seq data from various human tissues, both normal and cancer^35^. Along with standard RNA-seq data, TCGA provides miRNA-seq data from more than 1800 samples. Therefore, TCGA can provide a potential source of training data for investigating the miRNA landscape and regulatory network. Statistical data, such as read counts and normalized expression levels, are publicly available in both the GTEx and TCGA databases. In contrast, raw sequence data are available only for researchers authorized by the NIH Database of Genotypes and Phenotypes (dbGaP). A collaboration between TCGA consortium and the International Cancer Genome Consortium (ICGC) led to the Pan-Cancer Analysis of Whole Genomes (PCAWG) project. The sequencing effort included 2793 cancer patients across 20 primary sites, and RNA-seq data were obtained from 1222 patients^36^. These databases serve as important data sources when constructing training datasets for tasks involving differential gene expression regulation, eQTL, or cancer-associated gene expression regulation.

## Characteristics of RNA biology data and related deep learning methods

It is ideal to have a perfectly curated and sufficiently large dataset to train a DL model. However, obtaining such a dataset is not always possible, and adopting this approach is especially challenging in biology due to the relatively high cost of data production. Indeed, there have been multiple efforts to construct comprehensive biology databases, including GEO, SRA, and ENCODE, as reviewed in the previous section. However, such databases often suffer from limited metadata integrity^20,21^. Therefore, researchers often have to mitigate this issue when building training datasets for DL models in RB. A critical step in mitigating training dataset imperfection is to select an appropriate method for training DL models. Here, we review popular DL methods and describe their utility in leveraging RB data.

### Supervised learning

The term paradigm in machine learning corresponds to the classification of learning methods based on the input data and task^37^. Supervised learning utilizes labeled data to train a model to accurately predict the labels in the training set, whereas unsupervised learning trains a model to efficiently represent the input data without using labels. The combination of the two methods is termed semi-supervised learning, in which a model is simultaneously trained on large unlabeled datasets and small labeled datasets^38^. Supervised learning is the dominant choice among these paradigms since it can be directly applied to various downstream prediction tasks. Many predictive applications of DL in RB have also adopted supervised learning, e.g., miRNA target prediction, gene expression prediction, RBP binding prediction, ncRNA biogenesis prediction, and RNA modification prediction.

Supervised learning provides a straightforward framework for DL, but it is not without limitations. Supervised learning usually requires a sufficient amount of reliable labeled data, which is not always available, especially in RB^39^. Specifically, uncontrolled metadata in public RB databases impede the construction of labeled training datasets. Moreover, supervised learning can easily let DL models pick up biases in data labels, often introduced by experimental artifacts or data analysis pipelines. Even after standard filtering procedures, biological noise in experimentally generated data labels can complicate the training of DL models using supervised learning. Therefore, training a model to learn biological knowledge with supervised learning can occasionally be challenging. In such cases, exploring other DL paradigms can be beneficial.

### Self-supervised learning

Labeling data for supervised learning is often expensive and time-consuming, and the amount of labeled data is less than the amount of unlabeled data in most domains. This problem can be overcome by leveraging unsupervised learning. However, unsupervised learning methods are applicable only to limited types of RB tasks, such as cell clustering and substructure detection^40,41^. To address this limitation, a self-supervised learning paradigm has evolved. In self-supervised learning, a model is trained on unlabeled data as in unsupervised learning. However, the model is trained with a supervised learning objective by generating labels from unlabeled data itself^42,43^. This paradigm has gained momentum in natural language processing applications through the success of large language models^7,43^.

To employ self-supervised training in practice, a model is pre-trained on a large unlabeled dataset in a self-supervised manner, learning generalizable knowledge such as language structure. Then, the pre-trained model is fine-tuned to perform a specific downstream task using a smaller labeled dataset in a supervised manner. For example, scBERT was pre-trained on unannotated single-cell transcriptome data to predict the expression of randomly masked-out genes. Then, the model was fine-tuned to annotate and discover cell types^44^. Several other studies have applied a self-supervision framework for RB tasks, including disease modeling^45^, small molecule–miRNA association prediction^46^, and RBP prediction^47,48^. The success of these examples demonstrates the feasibility of learning biological context using massive amounts of unlabeled data. Therefore, self-supervised learning offers promising potential for deciphering the complex context of the transcriptome.

### Domain adaptation

Training DL models for RB is often obstructed by the scarcity of training data. This issue is not unique to RB^49^. Multiple DL methods have been proposed for resolving this issue. In some cases, the scarcity of training data in the desired domain can be overcome by leveraging data from other domains. Domain adaptation is a DL technique for such cases in which a model is trained to capture domain-invariant knowledge without using the target domain label^50,51^. In biology, the domain concept may correspond to different biological levels, organisms, cell lines, or batches of experiments. For example, scDEAL was initially trained with bulk-level cancer drug response prediction tasks using bulk RNA-seq data. Then, domain adaptation was employed to transfer the learned knowledge for single-cell-level drug response prediction tasks using scRNA-seq data^52^. Several other DL studies in RB have used domain adaptation methods to mitigate the scarcity of training data in the target domain, including isoform function prediction^53^, transcription factor (TF) binding site prediction^54^, and single-cell RNA-seq (scRNA-seq) data classification^55^. Therefore, domain adaptation can be useful in RB when generating training data is too costly, but related data in another domain are available.

### Meta-learning

Meta-learning, or ‘learning-to-learn’, is a collection of methods that allow a model to improve its ability to learn new tasks. In meta-learning, the model is trained on multiple tasks by iterating over each task to learn the knowledge that is generalizable to all the trained tasks as well as new tasks that it can encounter in the future. Meta-learning techniques are instrumental in few-shot tasks, where a model has to make predictions about classes that have only a few examples in the training set^56,57^. There have been efforts to employ meta-learning for RB since limited training data are a common issue. For example, MARS was trained on annotated and unannotated single-cell transcriptomes from multiple tissues. A meta-learning framework was employed to train a model that can annotate single-cell transcriptomes of novel tissues containing cell types that were not encountered during training^58^. Other successful examples of these methods include cancer survival prediction via gene expression^59^, ncRNA-encoded peptide (ncPEP) disease association prediction^60^, and lncRNA localization prediction^61^.

### Data augmentation

Data augmentation is a popular technique used in data-limited settings, in which diverse transformations are applied to input data to generate additional synthetic examples^62^. Ideally, the transformations should not affect the task outcome. The simplest forms of augmentation include cropping, rotation, flipping, resizing, recoloring, and blurring. However, such transformations are not always directly applicable to RB data. Instead, reverse complementing the nucleic acid sequence, shifting the sequence, or adding single-nucleotide insertions can be employed for RB data augmentation. Data augmentation has been used in various deep models for RB tasks, including coding potential prediction^63^ and gene expression prediction^54^.

### Ensemble

Ensemble learning is another technique that has been shown to improve the model performance when training data are limited. In ensemble learning, multiple DL models are combined via various methods, such as training multiple models with sampled data and voting, choosing the best model for each example, and training a model to combine the outputs of multiple models^64,65^. Multiple RB studies, including RBP motif prediction^66^, lncRNA identification^63,67^, splicing variant prediction^68^, and RNA modification detection via nanopore sequencing^69^, have leveraged ensemble techniques.

In summary, while supervised learning is a dominant paradigm of DL in RB, its utility is often limited by the availability of experimentally generated labels. To overcome this limitation, various methods, including self-supervised learning, domain adaptation, meta-learning, data augmentation, and ensemble, can be utilized. The potential of these methods can be leveraged for RB studies by selecting an appropriate method for a given task after analyzing the characteristics and imperfections of training data.

## Encoding RNA biology data into deep learning input features

DL models require numeric multidimensional arrays, termed tensors, as inputs to extract task-relevant features through matrix multiplication and nonlinear operations. However, many biological data are not provided in such forms. Thus, to utilize DL models in RB, it is crucial to transform these biological data into tensors through a process called encoding. Choosing a suitable encoding technique is cardinal to training a robust DL model. If an encoding method reflects too much information specific to a sample or an experiment, the DL model will fail to generalize. Conversely, the model will fail to learn if an encoding method removes too much information from the original data. Therefore, the essence of encoding is to efficiently represent the generalizable properties of the original data relevant to a given task. In the following, we present representative encoding techniques for RB data that have been successfully utilized in multiple studies.

### Nucleic acid sequences

**Fig. 1:**
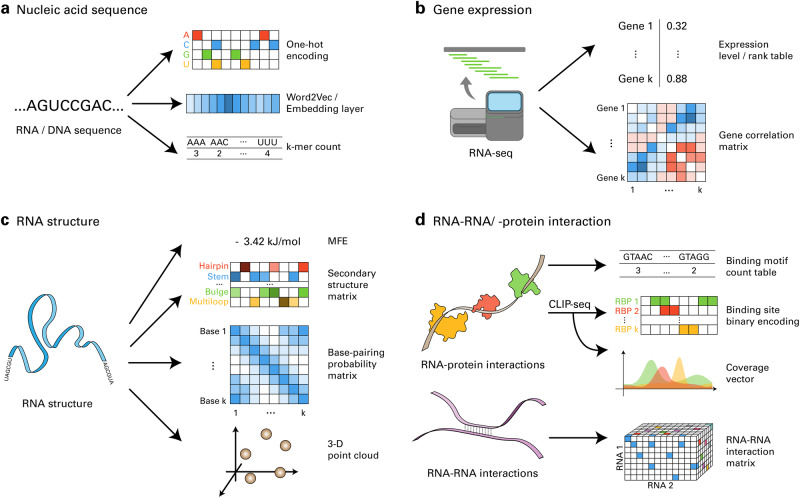
Processing RB data into input features of DL models. **a** Nucleic acid sequences can be encoded using one-hot encoding, word2vec, embedding layers, or k-mer counts. **b** When encoding the gene expression data, the normalized expression level or rank vector can be used as an input feature. Gene correlation matrices are also often used to capture gene‒gene interactions. **c** RNA structure data can be represented using the minimum free energy (MFE), secondary structure matrices, base-pairing probability matrices, or 3D point clouds. **d** RNA–protein interactions can be represented by counting known binding motifs or encoding CLIP results as binary binding site tensors or continuous coverage tensors. RNA‒RNA interactions can be encoded as 3D matrices to convey base pairing information.

Several types of biological data involving RNA can be leveraged to predict biological processes, including gene expression, RBP binding affinities, and regulatory elements. Among them, nucleic acid sequences are the most widely used data type since, in principle, numerous biological properties can be predicted solely from sequence data. The most common way of encoding nucleic acid sequences is to one-hot encode the bases, which may allow the DL model to automatically capture relevant features (Fig. 1a). In this simple one-hot encoding approach, each base is represented by a binary vector with a single 1, the position of which represents each nucleotide base. For instance, base A is encoded as [1, 0, 0, 0], and base C is encoded as [0, 1, 0, 0]. Moreover, some studies further improved simple mononucleotide one-hot encoding by integrating matrices corresponding to dinucleotides, trinucleotides, purines, pyrimidines, strong hydrogen bonds, weak hydrogen bonds, amino groups, and ketone groups^70,71^. Other studies have enriched the encoding through one-hot encoding of the k-mers in sliding windows^66^ and one-hot encoding of the pairwise alignment results^72^.

Although one-hot encoding is the most common method for encoding nucleic acid sequences, many studies have adopted techniques from natural language processing to produce more informative encodings of nucleotide sequences. By considering biological sequences as sentences and k-mers in the sequence as words, biological sequences can be encoded using the natural language encoding technique termed word2vec^73^. In word2vec, each word is represented by a large vector called a word embedding. A word embedding is learned by either predicting a word of interest from the surrounding words or predicting surrounding words from a word. Several studies have used word2vec to encode sequence data in RB to improve the performance^72,74–76^. Word2vec is generalized by expanding the subject from a word to a paragraph. This technique, named doc2vec^77^, was also adopted in RB to generate embeddings for the whole gene or miRNA sequences^78^. Word embeddings can also be generated during training time by randomly initializing embeddings and updating these vectors via a neural network layer called the embedding layer. This method can generate word embeddings that are more relevant to the objective of the model. For example, m6Anet employs this scheme to generate motif embedding vectors^79^. In addition to the encoding methods mentioned above, the numbers of nucleotide monomers and polymers were also used to encode sequence data^80,81^.

### Expressions

Expression data are important input features since the expression levels of regulatory genes and landmark genes are correlated with diverse biological processes. These data are primarily derived from RNA-seq and CAGE. The simplest method for encoding expression data is to use normalized expression values from RNA-seq data^82,83^ (Fig. 1b). Aptardi extended this simple encoding method by considering expression value fluctuations in a local region. Specifically, the authors partitioned the region into three equal-sized bins and calculated the differences in expression values between neighboring partitions^81^. Other studies, such as DMIL-IsoFun and HiCoEx, encoded global interactions of expression data with a 2-dimensional matrix^84,85^.

### Structures

RNA structures are fundamental data for investigating the interaction, functions, and stability of RNAs. Many DL methods utilize the RNA structure in various ways^80,82^ (Fig. 1c). The simplest way to encode the RNA structure is the minimum free energy (MFE)^86,87^. However, as a scalar value, the MFE conveys limited information, and DL models may benefit from more information-rich vector representations of RNA structures. There are several methods for encoding RNA structure data as a 1D tensor. For instance, the nucleotide-wise probability of structural contexts, such as hairpin loops, inner loops, and multiloops, can be used to obtain a continuous or binarized encoding tensor of RNA structures^88,89^. Furthermore, the frequency and distance of these structural contexts can be added to the encoding^86^. Apart from encoding structural contexts, the 3D coordinates of each atom or nucleotide in a tertiary structure can be encoded as a 1D tensor^90^. The RNA structure can be encoded as a 2D tensor of pairwise binding probabilities^72,85,91^.

### Bindings

Another type of data frequently used in DL for RB is the binding between RNA and regulatory molecules, including TFs, miRNAs, and RBPs, which play crucial roles in biological processes (Fig. 1d). One simple method for encoding protein‒RNA/DNA binding data is to count the known binding motifs in the region of interest^80,92^. However, this method does not accurately reflect the in vivo interactions between biological molecules. Several studies utilized in vivo binding data derived from CLIP data to address this issue. To encode the information from CLIP data, coverage vector^93^ and binarized binding matrix^94^ were used. Unlike protein–RNA interactions, RNA–RNA interaction data are often encoded as multidimensional tensors to convey base-pairing information. One way to encode miRNA‒target interactions is via a 3D matrix, in which the first two dimensions represent the miRNA and target sequence positions, and the last dimension represents the one-hot encoded combination of miRNA bases and target bases^95^.

## Deep learning architectures for leveraging RNA biology data

The DL architecture is the structure of neural networks, including connections between layers and operations in each layer. According to the universal approximation theorem, a neural network with a single sufficiently large hidden layer and a nonlinear function can approximate any continuous function^96^. However, such shallow architectures usually fall short in practical tasks, as they fail to learn or require too many neurons. Nevertheless, such problems have been alleviated with improved architectures. For instance, before the introduction of an efficient architecture named the convolutional neural network (CNN) by LeCun et al.^97^, DL was not widely applied to images due to the inefficiency and suboptimal performance. After it was introduced, the CNN architecture was widely adopted in computer vision tasks and exceeded human-level performance^6^. Therefore, choosing a suitable architecture that can work efficiently with a given data type and task is crucial. Here, we review DL architectures that have been shown to work effectively with various types of biological data and tasks.

### Multilayer perceptrons

**Fig. 2:**
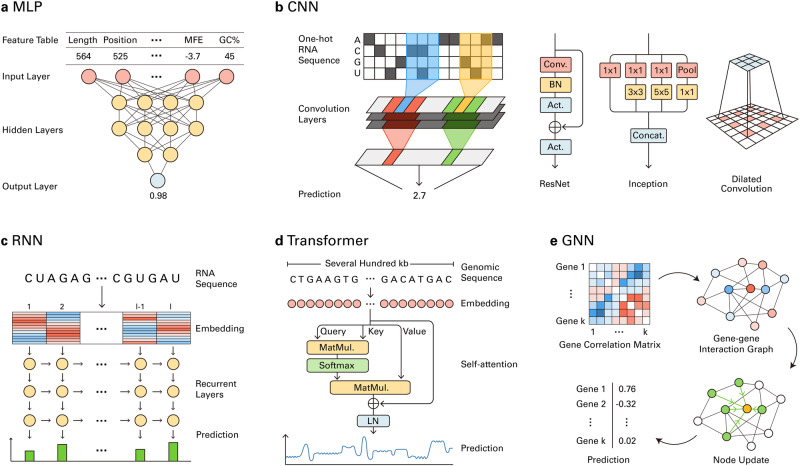
DL architectures for RB models. **a** MLPs can make probabilistic predictions from biological feature tables. **b** CNNs can predict biological features from one-hot-encoded biological sequences by capturing local patterns. ResNet, Inception, and dilated convolution can improve the performance and increase the input size. **c** RNNs can process embeddings of RNA sequences to provide basewise predictions of biological features. **d** GNNs can operate on gene–gene interaction networks derived from gene correlation matrices to predict genewise biological features. **e** Transformers can capture long-range interactions from genomic sequences of several hundred kilobases using multi-head self-attention.

The multilayer perceptron (MLP) is the simplest form of a DL model and comprises multiple hidden layers of neurons (Fig. 2a). Each neuron receives input from other neurons from the previous layer, performs linear transformation, and applies a nonlinear activation function. During training, the weight of each neuron is iteratively updated via backpropagation, which involves the computation of the gradient of the loss function for each training example^98^. MLPs can learn to generate informative vector representations of the input data, which allows them to perform predictive tasks such as regression and classification. Several early DL models in RB have been developed based on the MLP architecture for various tasks, including lncRNA prediction, gene expression prediction, tissue-specific alternative splicing prediction, and structure-based RBP binding site prediction^80,99,100^. Another neural network architecture similar to the MLP is the deep belief network (DBN), where each layer is initially trained individually and then trained together to construct and train a deeper neural network^101^. The DBN has been utilized for lncRNA identification^99^, tumor clustering^40^, and RBP binding site prediction^102^.

### Convolutional neural networks

The local patterns and interactions observed in tensor encodings of biological data, such as one-hot sequences, are fundamental features that are relevant to biological processes. For instance, the motifs for regulatory elements can be discovered by examining local interactions on DNA sequences, and RBP binding motifs can be discovered from local interactions of RNA sequences. Among DL architectures, CNNs have achieved robust performance in learning and aggregating local interactions^97,103^. Inspired by this success, CNN-based models have been frequently utilized for DL in RB. CNNs process data using weight matrices called convolution kernels, whose elements are randomly initialized (Fig. 2b). The core of the CNN is the convolution layer, which can be understood as the convolution kernels sliding across the input tensor. In each sliding step, the sum of the elementwise product for each kernel and each patch of the input tensor is calculated. By iterating through this process, the kernels can be updated to capture task-relevant local interactions in the input data. When biological sequences are used as inputs, convolution kernels can function as automatically learned motif detectors^104^. Hence, the natural use of CNNs is to detect local motif-related features such as regulatory elements^105–107^, protein–nucleic acid interactions^108^, nucleic acid–nucleic acid interactions^95^, 5′ UTR strength prediction^109^, and polyadenylation (poly(A))^70,110^. The CNN architecture has outperformed the classical machine learning and MLP architectures in various RB tasks. Several papers benchmarked the CNN and other architectures for specific RB tasks and demonstrated the robust performance of the CNN^111,112^.

Sophisticated biological properties require models to capture long-range interactions. This can be achieved by stacking additional convolution layers. However, simply adding more layers to the CNN results in suboptimal performance since the increased distance between the input and the output impedes the propagation of the gradient through the model. To overcome this limitation, He et al. proposed ResNet^6^, which utilizes skip connections to connect the input directly to the deeper layers. This architecture enables the stable training of the deep CNN models. ResNets have been used for DL in RB to capture longer interactions among captured motifs on biological sequences or data to predict splicing^113,114^, regulatory activities^105^, and N^6^-methyladenosine (m6A) modification^115^. ResNets can capture longer interactions than can simple CNNs but usually struggle to capture interactions between elements thousands of bases apart, such as splicing donors and receptor sequences. Therefore, several RB tasks, such as splicing prediction, require additional methods to capture long-range interactions. This problem can be addressed by adopting dilated convolution. In dilated convolution, each element of the kernel is applied to every n-th element of the input, increasing the range in which interactions can be captured^116^. Dilated convolution has been used in various RB tasks that require capturing long-range interactions^54,105,113,115^. In addition, integrating multiple convolution layers of different sizes, as suggested in the Inception architecture, can improve the performance of CNNs by allowing them to capture interactions of diverse sizes^117^. The idea was adopted in DeepExpression to predict gene expression from sequences^107^ and in CUP-AI-Dx to classify metastatic cancers using RNA-seq^118^.

### Recurrent neural networks

The natural analogy between a convolutional kernel and a motif detector has led many researchers to use CNNs to process biological sequence data. However, it is common in natural language processing to process sequence data with recurrent neural networks (RNNs)^119^. RNNs process input sequences recursively in order (Fig. 2c). In each recursion, the recurrent unit receives information from the prefix of the input sequence, processes the information, and passes it to the next unit. This recursive process results in the intrinsic ability of RNNs to capture interactions among sequence elements^120^, such as cis-regulatory elements. However, in practice, RNNs exhibit suboptimal performance when processing long input sequences. For long inputs, an extended number of recursion steps can lead to excessive accumulation or forgetting of past information, which leads to a failure to learn. Long short-term memory (LSTM) addresses this problem by automatically learning what proportion of information to forget or remember from the past sequence^121^. In RB, several studies have utilized LSTM or similar architectures, such as gated recurrent unit (GRU)^122^, to predict miRNA–gene associations^78^, poly(A) sites^81^, coding potentials^63^, and differential gene expression^123^. Notably, while most RNN models for natural language processing operate in a single direction, many RNN models for RB utilize bidirectional LSTM (BiLSTM)^124^ to exploit the bidirectional nature of genomic sequences and biological interactions. In addition, an RNN can be hybridized with a CNN, which, in principle, allows the CNN to detect local features and the RNN to capture higher-level interactions between captured features. This approach is also often used in RB for tasks such as alternative splicing prediction^125^, isoform function prediction^53^, RBP binding-altering variant identification^126^, and poly(A) site prediction^127^. A benchmark study demonstrated the superior performance of an RNN/CNN hybrid over a CNN or an RNN in RBP binding prediction^128^.

### Transformers

Both CNNs and RNNs have intrinsic limitations when processing sequence data: they can only capture long-range interactions indirectly through multiple layers or steps. This prevents CNNs and RNNs from learning the dynamic context of the sequence. To overcome these limitations, Vaswani et al. introduced the transformer architecture^129^. The transformer directly captures long-range interactions among sequence elements by using the self-attention mechanism (Fig. 2d). Self-attention captures every possible pairwise interaction between every sequence element using an attention matrix. Each weight of the matrix represents the relevance of each pairwise interaction to the task. During training, the weights of the attention matrix are updated to reflect the sequence context. Hence, transformers can better capture long-range interactions and dynamic contexts in sequences than CNNs and RNNs. Numerous studies have demonstrated the superiority of transformers in processing natural language, images^130^, and even biological data^8^. In RB, the transformer architecture has been applied to poly(A) signal prediction^131^, gene expression prediction^132^, cell type annotation^44^, network-level disease modeling^45^, and circRNA–miRNA interaction prediction^133^.

### Graph neural networks

Several RB data, including contact information, structure, coexpression, and base pairing data, can be represented as graphs instead of sequences. Representing biological data as a graph allows a flexible representation of interactions between biological entities. Graph neural networks (GNNs) capture information from such graph-type data. In a GNN, a vector corresponding to each node is updated by aggregating the information from connected nodes (Fig. 2e)^134–136^. The GNN can learn to capture interactions between nodes by repeating such updates^137^. In RB, GNNs have been applied to predict isoform function from isoform association graphs^84^, to predict RBP binding sites from graph representations of RNA secondary structures^91^, to integrate gene interaction networks and other biological networks^138^ and to predict gene coexpression from gene contact graphs derived from Hi-C data^85^.

## Applications of deep learning in RNA biology

**Fig. 3:**
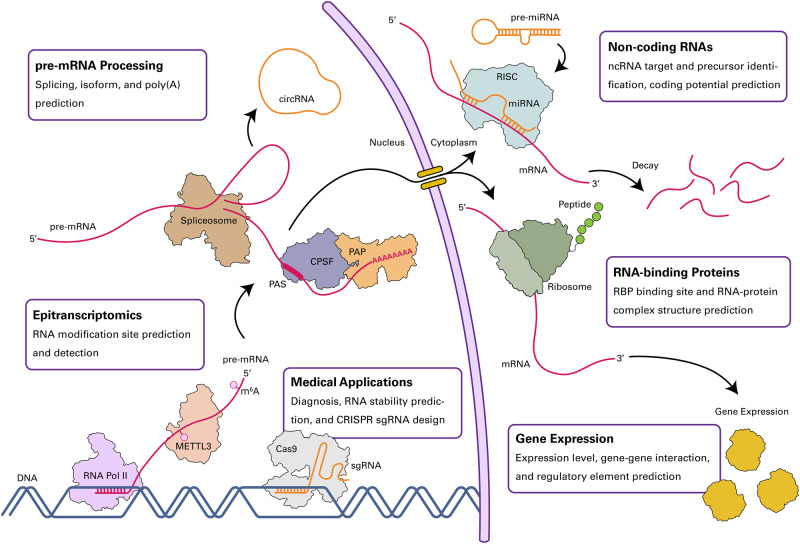
Applications of DL in RB. DL models provide insights into diverse biological processes involving RNA. In epitranscriptomics, DL models can predict and identify RNA modification sites. To understand pre-mRNA processing, DL models can predict alternative splicing and alternative polyadenylation sites, as well as isoform functions. In ncRNA biology, DL models can identify ncRNA targets, predict coding potential, and identify lncRNA precursors. To understand the biology of RBPs, DL models can predict RBP binding sites and protein‒RNA complex structures. To understand gene expression regulation, DL models can predict gene expression levels, coexpression, and regulatory elements. For medical applications, DL models can diagnose diseases, predict RNA degradation, and predict gene editing efficacy.

We have introduced crucial factors in developing DL models for RB, including large-scale data sources, encoding techniques, paradigms, and architectures. In this section, we demonstrate how these factors collectively contribute to building effective DL models for RB by reviewing specific examples. We review successful DL models that have achieved robust performance in important RB tasks or have showcased the potential of DL for biological discoveries (Fig. 3). By reviewing these models, we not only demonstrate the competence of DL in RB research but also suggest good practices for designing and training DL models that effectively leverage biological data.

### Noncoding RNAs

Noncoding RNAs (ncRNAs) are essential layers of transcriptional, posttranscriptional, and translational gene regulation and are crucial for tissue functions and developmental programs^139,140^. Diverse types of ncRNAs have been associated with cancer and genetic diseases^141,142^. One of the most representative types of ncRNAs is microRNAs, which are short RNAs produced from RNA hairpins. miRNAs are loaded into Argonaute (AGO) proteins and form RNA-induced silencing complexes (RISCs), which target mRNAs through complementary base pairing and reduce their expression^143^. The development of a model that can accurately predict the genome-wide regulatory effect of an arbitrary miRNA is a core task in RB that can enable a network-level understanding of miRNA-mediated gene regulation and pave the way for miRNA-based therapeutic development^144^. Several attempts to construct such a model using classical machine learning and DL algorithms have been made, underscoring the importance of the task. Among these models, TargetScan is the current state-of-the-art model. This model has achieved significantly improved accuracy in predicting miRNA targeting efficacy compared to that of previous machine learning methods based on thermodynamic models or correlative approaches^95^. TargetScan utilizes a CNN-based module to predict the affinities between an AGO–miRNA complex and target mRNAs. To alleviate the scarcity of experimental affinity data, the authors generated a large affinity dataset through AGO-RNA bind-n-seq (AGO-RBNS). The trained affinity predictor model was combined with a biochemical model to predict the repression of individual mRNAs. The model was more accurate than the previous versions of TargetScan, which did not use DL. Although TargetScan achieved robust performance in predicting miRNA targeting efficacy, it still explained only a small fraction of the variability in resulting mRNA expression changes from miRNA transfections. Therefore, this biologically important task needs further improvement, possibly through a more sophisticated approach to utilizing DL.

Long noncoding RNAs (lncRNAs) are ncRNAs longer than 200 nt. They bind to DNA, RNA, and proteins and regulate gene expression via various mechanisms, including chromatin remodeling, gene silencing and activation, nuclear organization, and RNA turnover regulation^139^. lncRNAs are transcribed from various sources, including intergenic regions, introns, antisense strands, and enhancers. Accurately distinguishing lncRNAs from mRNAs is an important challenge in RB but is often complicated by the presence of various isoforms^145^. Moreover, it is equally important to predict the functions of identified lncRNAs. To address both tasks, LncADeep utilizes separate modules for each task^99^. The DBN-based lncRNA identification module was trained on annotated transcript sequences to learn the features distinguishing lncRNAs from mRNAs. The MLP-based functional annotation module was trained on lncRNA‒protein interaction data from the NPInter database^146^. The functional annotations of the identified lncRNAs were made through pathway enrichment analyses of the predicted lncRNA-binding proteins via pathway databases, KEGG^147^ and Reactome pathway^148^. An average of 25 KEGG and 67 Reactome pathway annotations were associated with each identified lncRNA, indicating the complexity of lncRNA functions. The resulting model outperformed previous models in both tasks. While lncRNAs pose a unique challenge in RB due to their diverse regulatory mechanisms and sequence similarity to mRNAs, studies have shown that DL can be leveraged to elucidate the complex biology of lncRNAs.

Circular RNA (circRNA) is a subtype of lncRNA, which can accumulate in specific cell types due to the increased stability rendered by a closed ring structure. These accumulated circRNAs often act as sponges for miRNAs and RBPs, inhibiting their regulatory function and adding another layer of posttranscriptional gene regulation^149^. By modulating posttranscriptional gene regulators, circRNAs are associated with various diseases, such as cancer^150^. Therefore, identifying circRNAs is an important task. Distinguishing circRNAs from linear lncRNAs based on their sequences is challenging because the circularization process can often be inferred not from the transcript sequence itself but from the surrounding genomic context. This difficulty is reflected in the suboptimal performance of methods that distinguish circRNAs from other lncRNAs. To develop a more accurate circRNA identification model, circDeep uses reverse complement matching and conservation information from flanking regions of input sequences^74^. The circDeep architecture is a hybrid CNN-BiLSTM model that captures both local interactions and global interactions in input sequence features. This model was trained on 32,914 human circRNAs from the circRNADb^151^ and other lncRNAs from GENCODE^152^ to learn the features distinguishing circRNAs from other lncRNAs. Since circRNAs are not fully annotated in standard annotations such as GENCODE and Ensembl, it is necessary to refer to specialized databases such as circBase to construct circRNA-related training datasets^153^. The resulting model significantly improved the accuracy of the identification of circRNAs.

The utilization of DL to identify or distinguish major species of ncRNAs has significantly improved the accuracy compared to the previous machine learning methods (Table 2 and Supplementary Table 1). Nevertheless, functional annotation of identified ncRNAs still depends heavily on traditional enrichment analyses and thus remains a challenge for DL. To discover the specific functions or biological roles of ncRNAs, adequate DL methods, such as GNNs, which can learn from pathways or interaction networks involving ncRNAs, could be adopted.

**Table 2:** DL models for ncRNA biology.

Name	Input features	Task	Architecture	Paradigm	Ref.
DeepMirTar	Seed match, free energy, sequence composition, site location, site conservation, site accessibility, and RNA sequence (one-hot)	miRNA target prediction	Stacked denoising autoencoder	Unsupervised pre-training, Supervised fine-tuning	^87^
LncADeep	Manually selected sequence and homology features, minimum redundancy, maximum relevance selected sequence features, and structure features	lncRNA identification and lncRNA‒protein interaction prediction	DBN	Supervised	^99^
mRNN	RNA sequence (embedding layer)	Coding potential prediction	RNN (GRU)	Supervised	^63^
Aoki et al.	RNA pairwise sequence alignment (word2vec/one-hot) and RNA secondary structure (base-pairing probability matrix)	Classification of ncRNA pairwise alignments	CNN	Supervised	^72^
TargetScan 8	mRNA‒miRNA base-pairing matrix (one-hot)	miRNA‒target affinity prediction	CNN	Supervised	^95^
dnnMiRPre	RNA sequence and secondary structure combination (one-hot)	miRNA precursor prediction	CNN and LSTM	Supervised	^111^
circDeep	RNA sequence (word2vec), reverse complement matching, and conservation	circRNA identification	CNN-BiLSTM hybrid	Supervised	^74^
RNAsamba	RNA sequence and translated sequence (one-hot)	Coding potential prediction	CNN	Supervised	^67^
SG-LSTM-FRAME	miRNA sequence (Doc2vec), gene sequence (Doc2vec), miRNA network (Role2Vec), and PPI network (Role2Vec)	miRNA–gene relationship prediction	LSTM	Supervised	^78^
DeepLncPro	DNA sequence (one-hot, nucleotide chemical properties, and dinucleotide physical-chemical properties)	lncRNA promoter identification	CNN	Supervised	^71^
Finetuned DNABERT	RNA sequence (k-mer tokens)	circRNA–miRNA interactions prediction	Transformer	Self-supervised, supervised	^133^
DiMo	miRNA sequence (k-mer frequency and Word2vec)	miRNA motif identification	CNN and RNN (GRU and LSTM)	Supervised	^75^
GM-lncLoc	RNA sequence similarity graph	lncRNA subcellular localization prediction	GCN	Supervised, Meta-learning (MAML)	^61^

*PPI* protein‒protein interaction, *GCN* graph convolutional network, *MAML* model-agonistic meta-learning.

### Epitranscriptomics

Epitranscriptomics is a field of RB studying various RNA modifications, which are crucial components of posttranscriptional gene expression regulation. RNA modification affects splicing, poly(A), mRNA export, RNA degradation, and translation efficiency^154,155^. Through these biological processes, RNA modification is associated with cell differentiation, cancer progression, and neurological development^156,157^. Therefore, understanding the functions and regulatory mechanisms of RNA modification is a significant objective in RNA biology. Investigating the biology of RNA modifications requires profiling the deposition of RNA modifications at the transcriptomic scale. For this purpose, high-throughput experimental methods, including m6A-seq^11^, miCLIP^158^, and bisulfite-seq^159^, have been developed. Although these methods enable systematic investigations of epitranscriptomic landscapes, they often suffer from low resolution, high false positive rates, motif biases, and low concordance between experiments^160^. To overcome this limitation, researchers have applied DL methods to profile the epitranscriptomic landscape. Transcriptome-wide prediction of RNA modifications via DL can improve the understanding of posttranscriptional gene regulation by elucidating the underlying mechanisms, associated variants, and phenotypic effects of RNA modifications^161^.

One approach to this objective is to train DL models that predict RNA modification sites from transcript sequences. This approach has been applied to various RNA modifications, including m6A^115^, 5-methylcytosine (m5C)^162^, pseudouridine^163^, and 2′-O-methylation^164^. One notable example is iM6A, which learned contextual information around the experimentally validated m6A modification sites with a ResNet-based model^115^. The inputs were pre-mRNA sequences with 5000 flanking nucleotides, and the labels were generated based on m6A sites validated through m6A-CLIP experiments. The iM6A model outperformed classical machine learning methods in predicting m6A modifications and generalized well to the sites validated with m6A-label-seq, MAZTER-seq, m6ACE-seq, and miCLIP2. By analyzing the model, the authors hypothesized that m6A-associated variants accumulate 50 nt downstream of the m6A site. They validated this hypothesis by analyzing an independently generated experimental dataset. They also showed that previously known pathogenic single-nucleotide variants (SNVs) are associated with changes in m6A deposition and suggested that codon usage may affect m6A deposition. While many RNA modification prediction models focus on predicting a single type of modification, multiRM predicts twelve widely occurring modifications with a single model^76^. multiRM was trained on twenty epitranscriptome profiles generated from fifteen different technologies. The model utilizes three embedding schemes: a 1D convolution, a hidden Markov model, and word2vec. The embedding vectors generated by each scheme were fed into LSTM and attention methods to learn interactions among features relevant to different modifications. Analyses of attention matrix weights revealed modification motifs that were highly concordant with known RNA modification motifs. Moreover, the attention weights of different types of modifications were strongly correlated, indicating crosstalk among different modifications, as reported in previous studies. The above studies demonstrate the feasibility of elucidating the mechanisms regulating the epitranscriptomic landscape by developing DL models that predict the deposition of RNA modifications.

Another approach for applying DL to profile modification depositions is to develop a DL model that captures modification signatures from direct RNA sequencing (DRS) data. DRS is a technique in which native RNA is sequenced without the need for reverse transcription^165^. In this method, the electrical current changes during the translocation of an RNA molecule inside a nanopore are measured to infer nucleotide identity. Since canonical and modified RNA bases cause different electrical current shifts, DRS can be used to identify modified bases via DL^166^. m6Anet identifies m6A modifications on transcripts with DRS data^79^. m6Anet uses an embedding layer to encode 5-mers, and predicts the modification probability of each site using an MLP. The model first predicts the probabilistic measure for each read and then determines the site-level probability from these measures. This technique is called multiple instance learning. m6Anet performed better than previous methods and generalized well to other cell lines and species. In addition to m6A, Dinopore predicts adenosine-to-inosine (A-to-I) editing from DRS data^69^. Dinopore was constructed based on ResNet and uses multiple branches with various convolutional filter sizes to capture local interactions with different spans. This method outperformed previous methods and showed robust interspecies generalizability. As shown above, the development of DL models that accurately predict RNA modifications has enabled researchers to systematically study the regulatory elements relevant to RNA modifications and phenotypic or disease consequences of RNA modifications (Table 3 and Supplementary Table 2). Integrating diverse types of transcriptomic data, such as gene expression, RNA structure, and RBP binding data, will facilitate the development of multimodal DL models that learn systematic knowledge of the epitranscriptome, expanding the understanding of posttranscriptional regulation.

**Table 3:** DL models for RNA‒protein interactions.

Name	Input features	Task	Architecture	Paradigm	Ref.
deepnet-rbp	RNA sequence (count vector), secondary, and tertiary structure (one-hot)	RBP binding site prediction	RBM	Supervised	^102^
DeepBind	RNA sequence (one-hot)	RBP binding site prediction	CNN	Supervised	^104^
iDeepS	RNA sequence (one-hot) and RNA secondary structure (one-hot)	RBP binding site prediction	CNN-BiLSTM hybrid	Supervised	^174^
iDeepE	RNA sequence (one-hot)	RBP binding site prediction	CNN	Supervised	^66^
pysster	RNA sequence (one-hot), RNA secondary structure, and sequence combination (one-hot)	RBP binding site prediction	CNN	Supervised	^88^
DLPRB	RNA sequence (one-hot) and RNA secondary structure probability matrix	RBP binding site prediction	CNN and RNN	Supervised	^89^
Avsec et al.	RNA sequence (one-hot) and relative distance (spline transformation)	RBP binding site prediction	CNN with spline transformation	Supervised	^108^
RPI-Net	RNA sequence (one-hot) and adjacency matrix	RBP binding site prediction	GNN-BiLSTM hybrid	Supervised	^91^
DeepCLIP	RNA sequence (one-hot)	RBP binding site prediction	CNN-BiLSTM hybrid	Supervised	^126^
PrismNet	RNA sequence (one-hot) and RNA secondary structure probability matrix	RBP binding site prediction	CNN (ResNet and SENet)	Supervised	^176^
RoseTTAFoldNA	MSA of protein and nucleic acid sequences, template residue distance matrix, and cartesian coordinates of template	Protein–RNA complex structure prediction	Transformer	Supervised	^179^

*MSA* multiple sequence alignment.

### RNA-binding proteins

RNA-binding proteins (RBPs) control various aspects of gene expression regulation, including mRNA decay, mRNA-ncRNA interaction, RNA modification, translation efficiency, and RNA processing^167^. The main challenge in RBP biology is to model the binding preferences and predict the binding sites of RBPs, which are crucial for understanding posttranscriptional and translational regulatory mechanisms. To address this challenge, RBP binding prediction models have been commonly trained using experimental data from RNAcompete^168^ and CLIP-based experiments^9^, such as HITS-CLIP^169^, PAR-CLIP^170^, and eCLIP^171^. DeepBind is the foundational research for utilizing DL in RBP biology^104^. In DeepBind, a CNN motif detector predicts protein-binding motifs from genomic sequences, and the resulting motif feature vector is fed into feedforward layers to yield binding prediction scores. Each DeepBind model accounts for a single type of protein, and the models were trained for 194 RBPs. DeepBind achieved a robust performance and showed that the RBP motif preference knowledge learned from in vitro experiments can be generalized to in vivo transcriptomes via DL.

While DeepBind outperformed previous approaches using a motif detector, subsequent studies showed that integrating contextual information from RNA structure and sequences could improve RBP binding prediction. The biological rationale for this approach is that the binding affinity of RBPs for an RNA target is influenced not only by the local binding motif but also by the sequence composition and structural context of the target RNA^172,173^. Deepnet-RBP utilizes a multimodal DBN that receives primary, secondary, and tertiary structures^102^. The primary and secondary structures were encoded as k-mer count vectors, and the tertiary structures were encoded as structural motif indicating vectors. The secondary and tertiary structures were computationally predicted. The model outperformed classical machine learning models and predicted several potential secondary and tertiary structural motifs. Similarly, iDeepS utilized computational RNA structure prediction from RNAshape and slightly outperformed DeepBind^174^. However, computing the structures of RNA molecules is computationally burdensome and often inaccurate. Because of this limitation, iDeepE attempted to capture structural information from sequences surrounding the RBP binding sites^66^. Using two CNNs with local and global resolution filters, iDeepE outperformed previous DL models, including Deepnet-RBP.

While the above approaches use dense vector representations, molecular graphs are commonly used in biology and chemistry to model the structures of molecules, including RNA and proteins^134,175^. RPI-Net utilizes a GNN to learn the graph representation of the RNA structure and to predict RBP binding and outperforms previous DL models^91^. Moreover, the study pointed out that some CLIP techniques, including PAR-CLIP and HITS-CLIP, may introduce sequence bias in the training set and that previous machine learning and DL models picked up the bias. When building the training set for RPI-Net, the authors de-biased the PAR-CLIP data by replacing the biased nucleotides with random bases. This example shows the importance of inspecting and de-biasing biological data when training DL models.

PrismNet, one of the latest DL models for RBP binding site prediction, incorporates in vivo RNA secondary structure data produced using icSHAPE-seq instead of computationally folded structures^176^. In this study, the squeeze-and-excitation network (SENet) architecture, which captures global interdependencies, was employed with ResNet^177^. PrismNet achieved robust performance in the benchmark conducted by the authors and was applied to discover disease-associated SNVs that affect RBP binding. In Zhou et al., a CNN-based RBP binding prediction model trained using eCLIP data was utilized to predict the effect of noncoding variants on autism spectrum disorders (ASDs), underscoring the utility of RBP prediction in medical genomics^178^.

Overall, various studies have shown that it is possible to model the binding preferences and binding sites of RBPs accurately (Table 4 and Supplementary Table 3). The performance of RBP prediction models was improved by incorporating sequence context and in vivo structure information, highlighting the importance of non-motif features in RBP binding. Recent breakthroughs in protein structure prediction have enabled accurate prediction of protein‒RNA interactions at the structural level^179^. Plausibly, integrating protein‒RNA complex structure data with current RBP binding site prediction methods could assist in accurate RBP binding site prediction. Improvements in RBP prediction models will promote the discovery of drug targets and biomarkers since RBP binding is a cardinal component of posttranscriptional and translational gene expression regulation.

**Table 4:** DL models for epitranscriptomics.

Name	Input features	Task	Architecture	Paradigm	Ref.
Deep-2’-O-Me	RNA sequence (rna2vec)	2’-O-me site prediction	CNN	Supervised	^164^
pysster	RNA sequence (one-hot), RNA secondary structure, and sequence combination (one-hot)	A-to-I editing site prediction	CNN	Supervised	^88^
iPseU-CNN	RNA sequence (one-hot)	Pseudouridine site prediction	CNN	Supervised	^163^
Gene2vec	RNA sequence (one-hot, embedding layer, and word2vec), and neighboring methylation state encoding	m6A site prediction	CNN ensemble (voting)	Supervised	^300^
MultiRM	RNA sequence (one-hot, Conv1D, word2vec, and HMM)	Multiple RNA modification prediction	LSTM (attention weight)	Supervised	^76^
Dinopore	RNA sequence and signal from Nanopore DRS	A-to-I editing detection	CNN (ResNet)	Supervised	^69^
iM6A	Pre-mRNA sequence (one-hot)	m6A site prediction	CNN (ResNet)	Supervised	^115^
m6Anet	RNA sequence (embedding layer) and signal from Nanopore DRS	m6A site prediction	MLP	Supervised/MIL	^79^
Deepm5C	RNA sequence (one-hot, contextual one-hot, nucleotide chemical property and frequency, and word2vec)	m5C site prediction	CNN ensemble (stacking)	Supervised	^162^

*KO* knockout, *SNP* single-nucleotide polymorphism.

### Pre-mRNA processing

Pre-mRNA processing is a complex process involving 5′ capping, splicing, and 3′ poly(A). These processes serve as important points of posttranscriptional gene expression regulation, often through alternative splicing and alternative poly(A) (APA)^180,181^. Dysregulation of pre-mRNA processing can cause various genetic disorders, including muscular dystrophy and progeria^182,183^. A major task in this domain is the prediction of splicing, which will assist in the discovery of novel disease-associated splicing variants. This task can be further divided into exon usage prediction and splice site prediction. Exon usage prediction involves predicting the exon inclusion rate, termed the percent spliced in (PSI), using predefined exon boundaries^184^. The sequences and abundances of isoforms produced by alternative splicing inferred from RNA-seq data are commonly used for training and evaluation. Leung et al. were among the first to apply DL for alternative splicing prediction. They utilized MLP to predict tissue-specific alternative splicing from manually extracted genomic sequence features and one-hot encoded tissue types^80^. In this study, alternative splicing prediction was formulated as a classification task using only three PSI categories—low, medium, and high—and therefore, the predictive capability was limited. In their subsequent work, Xiong et al. regarded the PSI as a continuously distributed variable^92^. Using this strategy, the authors discovered novel ASD-associated splice variants and inferred the pathogenic mechanism of specific SNVs in Lynch syndrome.

Splice site prediction involves the identification of splice sites from the genomic sequence. SpliceRover employs a CNN motif detector to predict splicing donor and acceptor site probabilities using genomic sequences of 15–402 nucleotides and outperformed previous machine learning methods^185^. This model was analyzed, and it was found that the model not only detects well-known splice site motifs but also considers additional factors, such as the polypyrimidine tract. SpliceAI adopts dilated convolution kernels to utilize longer sequence contexts of up to 10,000 nucleotides^113^. The authors showed that capturing long-range contexts improved splicing site prediction by comparing the performances of models with varying input lengths. The SpliceAI prediction score was used to discover a novel type of splicing variant, termed the cryptic splice variant, which creates splicing sites weaker than canonical splice site variants. In contrast to canonical splice variants that exert similar effects across tissues, cryptic splice variants alter splicing in a tissue-specific manner and are associated with intellectual disability and ASD. This example underscores how developing and utilizing DL models can assist novel scientific discoveries in RB.

Predicting poly(A) is another major task for DL in mRNA processing since APA is an essential regulatory mechanism of differential gene expression and is associated with various diseases. Poly(A) position data from PolyA-Seq^186^ and 3P-seq^187^ are commonly used to train poly(A) prediction models. DeepPolyA and Leung et al. are initial works that adopted different strategies to utilize DL for poly(A) prediction from genomic sequences^112,188^. DeepPolyA formulates this task as a binary classification problem between poly(A) sites and non-poly(A) sites, and Leung et al. formulate the task as a regression of the poly(A) site strength. Both models outperformed classical machine learning approaches for predicting poly(A) sites. While these models were trained from an in vivo dataset, a subsequent CNN model, APARENT, utilizes a large in vitro dataset generated by massively parallel reporter assay (MPRA) to represent biological complexity^189^. APARENT formulated the task as isoform fraction regression and cleavage site distribution prediction. Using APARENT, it was possible to capture several determinants of poly(A) site selection, identify potential pathogenic poly(A)-affecting variants, and design poly(A) signals that produce desired isoforms. The success of APARENT has demonstrated the utility of massive-scale experiments in developing DL models for understanding complex biological processes. Overall, multiple studies have demonstrated the utility of DL in discovering regulatory sequences and context features that determine pre-mRNA processing (Table 5 and Supplementary Table 4).

**Table 5:** DL models for pre-mRNA processing.

Name	Input features	Task	Architecture	Paradigm	Ref.
Leung et al.^80^	1393 manually selected sequence features and tissue index (one-hot)	Splicing (PSI) prediction	MLP and Bayesian inference	Supervised	^80^
Xiong et al.	1393 manually selected sequence features	Splicing (PSI) prediction	MLP ensemble and Bayesian inference	Supervised	^92^
Jha et al.	1357 manually selected genomic features, 874 manually selected CLIP features, tissue index, and knockdown/overexpression index	Splicing (PSI) prediction	MLP autoencoder	Supervised	^94^
Leung et al.^112^	DNA sequence (manually selected features, one-hot)	Poly(A) site strength prediction	MLP and CNN	Supervised	^112^
DeepPolyA	DNA sequence (one-hot)	Poly(A) site prediction	CNN	Supervised	^188^
SpliceRover	DNA sequence (one-hot)	Splice site prediction	CNN	Supervised	^185^
DeepIsoFun	Gene expression matrix and isoform expression matrix	Isoform function prediction	MLP autoencoder	Supervised	^53^
COSSMO	DNA and RNA sequence (one-hot) and intron length	Splice site prediction	CNN-LSTM hybrid	Supervised	^125^
Avsec et al.	RNA sequence (one-hot) and relative distance (spline transformation)	Splice branchpoint prediction	CNN with spline transformation	Supervised	^108^
DeeReCT-PolyA	DNA sequence (one-hot)	Poly(A) signal identification	CNN	Supervised	^110^
DeepGSR	DNA sequence (mono-, di-, and tri-nucleotide one-hot)	Poly(A) signal and TIS identification	CNN	Supervised	^70^
DeepPASTA	RNA sequence (one-hot) and RNA secondary structure (one-hot)	Poly(A) site prediction	CNN-BiLSTM hybrid	Supervised	^127^
APARENT	DNA sequence (one-hot)	proximal APA isoform prediction	CNN	Supervised	^189^
DARTS	exon-specific cis sequence and RBP expression	Differential alternative splicing between samples	MLP	Supervised	^82^
SpliceAI	Pre-mRNA sequence (one-hot)	Splice site prediction	CNN (ResNet, WaveNet, and dilated convolution)	Supervised	^113^
MMSplice	DNA sequence (one-hot)	Splicing variant effect prediction	CNN	Supervised	^68^
SANPolyA	DNA sequence (one-hot)	Poly(A) signal identification	Transformer	Supervised	^131^
Aptardi	Features extracted from DNA and RNA sequences	Sample-specific Poly(A) site prediction	BiLSTM	Supervised	^81^
DMIL-IsoFun	Isoform sequences (embedding layer) and coexpression data	Isoform function prediction	CNN, GCN	Supervised	^84^
Pangolin	DNA sequence (one-hot)	Splice site prediction	CNN (ResNet and dilated convolution)	Supervised	^114^

*GCN* graph convolutional network.

### Gene expression

One fundamental goal in genomics and RB is to model gene expression regulation using computational models. This goal is often formulated as predicting the gene expression level given the genomic sequence^190^. Solving this task would allow a comprehensive and quantitative analysis of the regulatory functions of noncoding sequences and the prediction of noncoding variant effects in silico. Despite its importance, the complexity of the mechanisms regulating gene expression has impeded conventional machine learning models from solving this task. Consequently, multiple studies have utilized DL to address this fundamental task.

Basenji and ExPecto are pioneering works on this topic^105,191^. Both are CNN models that predict the expression level of a given genomic sequence. Basenji has a receptive field of 32 kb and utilizes dilated convolution to predict the results of DNase-seq, ChIP-seq, and CAGE jointly. Among them, CAGE corresponds to gene expression. Unlike Basenji, ExPecto uses a sequential approach in which the first module of the model predicts epigenetic markers and TF binding from 40-kb DNA sequences, and the downstream modules predict gene expression levels. The epigenetic module was trained with ENCODE and Roadmap Epigenomics data, while the expression module was trained with CAGE data. Basenji and ExPecto both yielded gene expression level predictions that agreed well with the experimental eQTL data. ExPecto was also utilized to predict disease risk alleles and to predict and prioritize causal genome-wide association study (GWAS) variants. Xpresso is another CNN developed to predict gene expression levels from sequences. Unlike Basenji and ExPecto, Xpresso did not utilize epigenetic data for training and strictly relied on the genomic sequence^192^. The authors showed that the sequence-only model can perform comparably to previous models using epigenetic features, suggesting that gene expression can be inferred from flat genetic sequences. Moreover, Xpresso exhibits robust generalizability between cell lines and species. Notably, Xpresso was utilized to derive a novel hypothesis that CpGs are enriched in the core promoters of highly expressed genes, potentially expanding the role of CpG in transcriptional regulation and emphasizing that DL models can aid in novel biological discoveries.

In contrast to the models mentioned above, which all use the CNN architecture, Enformer was built with the transformer architecture to effectively capture long-range interactions between regulatory elements^132^. The input length for this model is 200 kb, which is significantly longer than that of the previous models. This model outputs TF ChIP-seq, histone modification ChIP-seq, DNase-seq, ATAC-seq, and CAGE tracks only from genomic sequences. Enformer outperformed Basenji2^54^ and ExPecto in RNA expression prediction. In addition, this model exhibited robust eQTL variant effect prediction and mutation effect prediction performance. The study verified the benefit of using the attention layer through an ablation study, demonstrating the effectiveness of transformers for processing long biological sequences. Moreover, Enformer showed the possibility of detecting candidate enhancers using attention weights and gradient-based saliency scores. Overall, multiple studies have shown that inferring gene expression solely from genomic sequences is possible using DL. This trend suggests that utilizing longer genomic sequences with an effective architecture would lead to better sequence-based modeling of gene expression.

Although sequence-only approaches have been successful in modeling gene expression, researchers have modeled gene expression based on non-sequence features, which could expand the dimension of gene expression regulation research. D-GEX is an MLP model developed to predict gene expression using the expression levels of 1000 landmark genes, enabling whole-transcriptome profiling via economic Luminex beads^83^. GEARS is another DL model for learning gene–gene interactions^193^. In GEARS, the relative change in gene expression upon perturbation of specific genes was predicted using an MLP. DeepChrome is a CNN that predicts gene expression levels from histone modification profiles, and it outperformed classical machine learning models for histone-based expression prediction^194^. DEcode is a CNN that infers differential gene expression levels from the binding information of three types of regulatory molecules, RBPs, miRNAs, and TFs. Using these input features allowed modeling at both the transcriptional and posttranscriptional regulation levels^195^. The output of DEcode is a tissue-wide expression profile of each gene, composed of relative gene expression levels in 53 human tissues. The authors analyzed the model to measure the importance of each regulatory molecule in the differential expression for each tissue. The regulatory significance of RBPs, miRNAs, and TFs inferred by analyzing DEcode was validated with in vivo loss-of-function mutation data and disease associations. This example shows how a DL model can prime the production of new biological knowledge.

Translational regulation is another important axis of gene expression regulation that can be probed by ribosome profiling and protein reporter assays. The sequence and structure of the 5′ UTR are the key determinants of translational regulation. Optimus 5-Prime utilized a CNN to predict ribosome load from the 5′ UTR sequence using the MPRA dataset for training^196^. Optimus 5-Prime accurately predicted the effect of the 5′ UTR sequence on the ribosome load. An analysis of the model revealed that the model can capture translation initiation site (TIS) sequences, stop codons, and non-canonical start codons. The model was also utilized to design a 5′ UTR of desired strength, demonstrating how such DL models can be utilized for synthetic biology. TISnet is a DL model for TIS prediction from primary sequences and RNA structures, the architecture of which was adopted from PrismNet^197^. The study showed that both sequence and structure contribute to the accurate prediction of the translation initiation probability of a given AUG. By analyzing the downstream regions of predicted start codons, it was hypothesized that the downstream hairpin structure dictates start codon selection. This hypothesis was experimentally validated, providing a critical point for rational protein design.

Overall, several studies have shown that DL can be leveraged to model gene regulatory mechanisms at the transcriptional, posttranscriptional, and translational levels (Table 6 and Supplementary Table 5). These DL models for gene expression modeling have paved the way for in silico identification of pathogenic variants, potential biomarkers, drug targets, and synthetic biology.

**Table 6:** DL models for gene expression.

Name	Input features	Task	Architecture	Paradigm	Ref.
DeepChrome	Histone modification matrix	Gene expression prediction	CNN	Supervised	^194^
D-GEX	Expression of landmark genes	Expression of target genes	MLP	Supervised	^83^
Cuperus et al.	5′UTR sequences (one-hot)	Gene expression prediction	CNN	Supervised	^109^
ExPecto	DNA sequence (one-hot)	Gene expression prediction	CNN	Supervised	^191^
DeepDiff	Histone modification matrix	Differential Gene expression prediction	LSTM-Attention hybrid	Supervised	^123^
Basenji	DNA sequence (one-hot)	Gene expression prediction	CNN (Dilated convolution)	Supervised	^105^
Optimus 5-Prime	UTR sequence (one-hot)	Gene expression prediction (mean ribosome load)	CNN	Supervised	^196^
DeepExpression	DNA sequence (one-hot)	Gene expression prediction	CNN (Inception)-LSTM hybrid	Supervised	^107^
DEcode	Experimentally determined binding sites of 762 TFs and 171 RBPs; predicted binding sites of 213 miRNAs	Differential gene expression prediction	CNN	Supervised	^195^
Basenji2	DNA sequence (one-hot)	Gene expression prediction	CNN (Dilated convolution, ResNet)	Supervised	^54^
Xpresso	Promoter sequence (one-hot) and sequence features correlated with mRNA decay	Gene expression prediction	CNN	Supervised	^192^
Enformer	DNA sequence (one-hot)	Genomic track prediction	Transformer	Supervised	^132^
TFCNN	TF binding matrix	Gene expression prediction	CNN	Supervised	^93^
HiCoEx	Gene contact network	Gene coexpression prediction	GNN (graph attention)	Supervised	^85^
TISnet	RNA sequence (one-hot) and RNA secondary structure	Classify initiating and non-initiating AUGs	ResNet and squeeze-excitation (PrismNet)	Supervised	^197^
GEARS	Coexpression graph and GO graph	Multigene perturbation outcome prediction	GNN	Supervised	^193^
LegNet	DNA sequence (one-hot), strand, and singleton	Gene expression prediction	CNN (EfficientNet)	Supervised	^248^

*GO* gene ontology.

### Medical applications of RNA biology

Fundamental discoveries in RB have made multiple contributions to medicine, including RNA vaccines^198^, RNA-targeting drugs^199,200^, and RNA therapeutics^201,202^. Numerous studies have also shown that transcriptomic data can be leveraged for disease diagnosis^203,204^. By replacing or complementing conventional methods, RNA-seq can be utilized for precise diagnosis and to provide personalized treatment strategies. For example, Mayhew et al. aimed to detect acute infection from the expression of 29 marker host genes^205^. Although it is theoretically possible to diagnose infection using the host transcriptome, this approach has been impeded by the transcriptomic heterogeneity between patients. To overcome this limitation, the authors trained an MLP named IMX-BVN-1 using an infection dataset compiled from GEO and ArrayExpress. The model successfully diagnosed infections in an independent ICU dataset. This example shows that using a predefined set of marker genes allows the efficient development of a diagnostic model. However, the limited availability of comprehensive marker gene sets often obstructs this approach for various diseases. Moreover, utilizing the entire transcriptomic landscape, instead of using only a small fraction of the transcriptome, may unleash the potential of DL. For example, Comitani et al. developed a DL model for pediatric cancer classification from the expression of 18,010 genes and pseudogenes profiled by RNA-seq^206^. Transcriptional diversity in pediatric tumor tissue has been a major issue in transcriptome-based diagnosis. The authors adopted a self-supervised learning framework to overcome this challenge. First, they developed RACCOON, a framework for unsupervised tumor transcriptome clustering and identification. The tumor subtype hierarchy output by RACCOON was utilized to train OTTER, an ensemble of multiclass classifier CNNs. The accuracy of pediatric cancer type prediction by OTTER was 89%, and the predictions were temporally consistent. These examples underline the capability of DL models to generalize from copious amounts of high-dimensional transcriptomic data to yield clinically valuable predictions.

Multiple studies have developed DL models for clinical diagnosis by integrating transcriptomic data with other modalities, including genomic and proteomic data. This multimodal approach improves performance and generalizability by allowing models to generate more comprehensive representations of the biological states of patients. MOGONET is a multimodal and multiomics GNN for patient classification that integrates DNA methylation, mRNA expression, and miRNA expression profiles^207^. In MOGONET, separate GNNs generate initial prediction vectors from each of the three inputs, and the tensor product of the three prediction vectors is passed to an MLP for patient classification. MOGONET was validated against an Alzheimer’s disease diagnosis task, a glioma grade classification task, a kidney cancer type classification task, and a breast invasive carcinoma classification task. In addition to direct diagnosis, multimodal DL has also been utilized for biomarker discovery, a crucial component of diagnostic development. As another example, CoraL is a multimodal CNN that predicts disease associations of ncRNA-encoded small peptides (ncPEPs) and their originating short ORFs (sORFs) for cancer biomarker discovery (59). The model was trained with meta-learning and demonstrated the generalizability of DL-powered cancer biomarker discovery across various types of cancers. HE2RNA is another multimodal DL model for clinical RB that predicts the gene expression profile from histology images^208^. HE2RNA is an MLP that generates a patch-level transcriptomic representation from whole-slide images of tumor tissues. The gene expression levels were obtained from the TCGA database. In addition to predicting gene expression levels, HE2RNA can also generate spatial gene expression maps and predict microsatellite instability via transfer learning. These examples show that transcriptomic data can be integrated with other data modalities through DL to produce medical predictions.

In addition to diagnosis, transcriptomic data have also been leveraged for prognosis prediction via DL. For example, Qiu et al. utilized RNA-seq data from TCGA to train an MLP that predicts patient survival from the expression level of 17,176 genes^59^. The authors adopted a meta-learning strategy to learn the parameter initialization, which allowed a few-shot training of the final model. This study demonstrated the utility of meta-learning for the few-shot survival prediction task by comparing it with regular pre-training and combined learning. Moreover, the feasibility of few-shot learning in transcriptome-based prognosis prediction was validated by demonstrating that the performance of the few-shot model was comparable to that of a many-shot model.

The success of mRNA vaccines amid the COVID-19 pandemic^209^ has drawn the attention of the pharmaceutical industry to RNA-based drugs. Beyond COVID-19 vaccines, RNA-based drugs offer promising therapeutic potential for preventing infections and treating various diseases, including cancer, cardiovascular diseases, and neurodegenerative diseases^210^. DL has also been utilized for RNA-based drug research since it has been widely used in pharmaceuticals to aid in drug target discovery and drug design^211,212^. A central task in developing effective mRNA-based drugs is to improve their stability, which is often a limiting factor in the global distribution of mRNA vaccines. Stanford OpenVaccine is a crowdsourced effort to develop a DL model that accurately predicts the stability of an arbitrary mRNA molecule^213^. The project was hosted on Kaggle, a Google platform for public DL competitions. The best solution outperformed previous machine learning models using data augmentation and ensemble^214^.

Gene editing, which has recently been approved for clinical use^215^, is another major field in RNA-based drug research. Several DL methods have been developed for CRISPR‒Cas editing systems, focusing on tasks such as sgRNA optimization and editing outcome prediction. DeepCRISPR utilizes unsupervised representation learning to train a denoising CNN that learns the representation of sgRNAs from sequence and epigenetic features^216^. The model was fine-tuned to predict on- and off-target effect profiles of sgRNA in the CRISPR-SpCas9 knockout system. CRISPRon exhibited robust performance in gRNA efficiency prediction by training a CNN that uses protospacer and protospacer adjacent motif (PAM) sequences as input^217^. For CRISPR-based base editing, BE-DICT, a transformer-based encoder–decoder model, was developed to predict the probabilistic outcomes of CRISPR-SpCas9 base editors given the protospacer sequence^218^. In addition to CRISPR-Cas9, DL-based guide RNA optimization tools have been developed for CRISPR-Cas13d and prime editing^219,220^. Similarly, RNAi drugs such as short hairpin RNAs (shRNAs) have been employed in RNA therapeutics. The mechanism of shRNA drugs is based on the repression of target gene expression through complementary base pairing between the shRNA and its target mRNA. Embedding shRNAs into miRNA backbones greatly increases the degree of target gene repression due to the utilization of the endogenous miRNA processing pathway, and these miRNA-embedded shRNAs are referred to as shRNAmirs. Predicting the targeting efficacy of shRNAmirs remains a key step in designing RNAi drugs based on shRNAmirs. In this context, deep learning models that predict the efficacy of shRNAmirs have been developed, and one CNN-based model named shRNAI+ has achieved robust performance by using sequence and context features^221^. These studies suggest that DL can be utilized for designing and optimizing RNA-based therapeutics.

Another important avenue in medical applications of RNA is RNA-targeting drugs. Gao et al. utilized DL to discover therapeutic targets for the splice-correcting drug BPN-15477^222^. They first selected potentially pathogenic splicing-altering mutations as candidate targets using SpliceAI. To identify drug targets among the candidates, they developed a CNN that predicts drug-induced changes in PSI, given the exon‒intron boundary sequences. Several targets predicted by the CNN were experimentally validated, providing potential therapeutic strategies for diseases, including Lynch syndrome, cystic fibrosis, and Wolman disease. Overall, numerous studies have demonstrated the merit of DL in various medicine-related RB tasks, positioning DL as a core asset in personalized and precision medicine (Table 7 and Supplementary Table 6).

**Table 7:** DL models for medical applications of RB.

Name	Input features	Task	Architecture	Paradigm	Ref.
Young et al.	Gene expression level of selected genes	Tumor clustering	MLP (RBM and DBN)	Unsupervised	^40^
DeepCRISPR	DNA sequence (one-hot), epigenetic marks (CTCF binding, chromatin-opening, H3K4me3 position, and DNA methylation information) as binary vectors	CRISPR sgRNA on-target and off-target knockout efficacy prediction	CNN	Unsupervised, supervised	^216^
DeepSEA (extended)	Chromatin profiles (histone marks, TFs, and DNase1 profiles) and RBP profiles	Prediction of disease impact of variants	CNN	Supervised	^178^
CUP-AI-Dx	Gene expression	Tumor primary site prediction	CNN (1D inception)	Supervised	^118^
HE2RNA	Whole-slide image (feature extraction using CNN (ResNet))	Gene expression prediction	MLP	Supervised (weakly supervised)	^208^
Qiu et al.	RNA sequencing data	Genomic survival analysis (survival time prediction)	MLP	Meta-learning, supervised	^59^
IMX-BVN-1	Expression values of selected 29 mRNAs	Acute bacterial and viral infection classification	MLP	Supervised	^205^
CRISPRon	Target‒DNA sequence (one-hot) and gRNA target‒DNA binding energy	gRNA efficiency prediction	CNN	Supervised	^217^
MOGONET	Multiomics data (mRNA expression, DNA methylation, and miRNA expression)	Classification of Alzheimer’s Disease, low-grade glioma, kidney cancer, and breast invasive carcinoma	GCN-MLP	Supervised	^207^
BE-DICT	Protospacer sequence	Base editing outcome prediction	Transformer	Supervised	^218^
Gao et al.	RNA sequence (one-hot for each exon-triplet)	Prediction of splicing changes after BPN-15477 treatment	CNN	Supervised	^222^
Wayment-Steele et al.	RNA sequence, minimum free energy, RNA secondary structure, and base-pair probability matrix	RNA degradation prediction	Crowdsourced models, including autoencoder, GNN, GRU, and CNN	Supervised	^213^
scDEAL	Cell line gene expression matrix and Single-cell gene expression matrix	Single-cell level cancer drug response prediction	Denoising autoencoder and domain-adaptive neural network	Supervised, transfer learning	^52^
TIGER	gRNA sequence (one-hot), target‒DNA sequence (one-hot), minimum free energy, target accessibility, target location, and gRNA secondary structure	gRNA efficiency prediction	CNN	Supervised	^220^
PRIDICT	Original and edited RNA sequences; 67 predefined sequence features, including minimum free energy and melting temperature.	Prime editing efficiency prediction	RNN (bidirectional, attention-based)	Supervised	^219^
CoraL	Peptide sequence	Cancer-associated ncPEPs prediction	CNN	Supervised, contrastive, meta-learning	^60^
OTTER	Gene expression matrix	Pediatric cancer classification	CNN Ensemble	Supervised	^206^
shRNAI+	Sequences of gRNA sequence or gRNA target site and surrounding regions (one-hot); in vitro processing efficiency.	shRNAmir efficacy prediction	CNN	Supervised	^221^

*RBM* restricted Boltzmann machine, *DBN* deep belief network, *GCN* graph convolutional network.

## Desiderata for employing deep learning for RNA biology

**Fig. 4:**
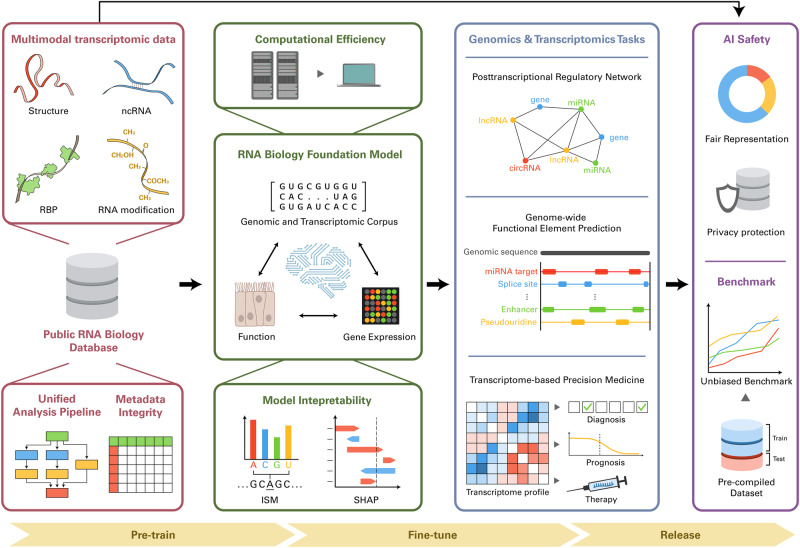
Desiderata for employing DL in RB. First column: Integrating multimodal transcriptomic data and constructing well-curated databases are prerequisites for developing effective DL models for RB. Unified analysis pipelines and controlled metadata are desirable properties of public RB databases. Second column: Foundation models for RB can be pre-trained with genomic and transcriptomic corpora, functional genomics data, and gene expression data. These models can learn the generalizable knowledge of genetic systems and language. Employing techniques for enhancing the computational efficiency and interpretability of DL models would further improve the utility of DL in RB. Third column: The RB foundation model can perform a wide range of downstream tasks after fine-tuning, including comprehensive network analysis of the posttranscriptional regulatory network, genome-wide prediction of functional elements, and transcriptome-based precision medicine tasks. Fourth column: The released DL model should comply with safety standards, which can be achieved by ensuring fair representation of the population and protecting the privacy of study participants in the training dataset. Moreover, the released model should be benchmarked using standardized pre-compiled datasets. Parts of this figure were created with BioRender.com.

Numerous examples of successful DL applications in RB demonstrate the competence of DL in elucidating the mechanism of biological processes from large-scale experimental data. Yet, significant challenges remain. First, the imperfect metadata integrity of databases and the scarcity of independent benchmarks hinder the training of effective DL models. Indeed, most DL models in RB have not reached expert-level or experiment-level performance. Second, the difficulty in understanding the prediction of models often impedes the achievement of biological discoveries via DL. Third, the models should be able to integrate diverse modalities of RB data to gain a systematic understanding of the transcriptome. Nevertheless, researchers are actively addressing these challenges. The collective efforts of institutions and laboratories are improving the availability and quality of public RB data. Researchers are adopting novel techniques and integrating multiple modalities to construct models that can perform a wider range of tasks than previous models. In this section, we review the desiderata for exploiting the full potential of DL for RB research, together with prominent DL techniques and efforts to meet the challenges (Fig. 4).

### Multidimensional and well-curated databases

The generation of structured and labeled datasets is central to developing DL models for RB. These datasets are commonly constructed from public databases containing high-throughput experiment data. Therefore, constructing public databases that reflect various dimensions of the transcriptome is cardinal in developing DL models for the integrative and systematic study of the transcriptome. These dimensions include functional genomics, isoforms, and single-cell transcriptomics. As reviewed in the previous section, ENCODE is a representative effort to construct a large-scale functional genomics database^223^. The ENCODE database is expanding to capture more diverse aspects of the transcriptome and genome. The current phase of ENCODE (ENCODE4) primarily focuses on increasing the diversity of TFs, RBPs, and cell types assayed. Moreover, to profile isoform expression across various cell types, long-read sequencing data, such as PacBio and Oxford Nanopore sequencing data, are included in the current phase. The efforts by ENCODE to increase the breadth and dimensions of the transcriptomic data have improved the utility of ENCODE for RB researchers. Single-cell transcriptomics is one of the most rapidly growing fields in RB, which allows spatiotemporal dissection of gene expression and regulation at the single-cell level. The Human Cell Atlas (HCA) is a representative single-cell transcriptomics database that aims to construct reference single-cell transcriptomes of diverse healthy human and mouse cells. The database currently contains data from more than 50 million cells^224^. The HCA has played a vital role in early research during the COVID-19 pandemic, illuminating cell- and tissue-level pathology^225^. These public databases that increase the dimension of RB data provide core assets for developing DL models in RB.

As reviewed in the previous sections, the metadata integrity and quality control (QC) of public databases are critical for filtering and labeling data during DL dataset construction. However, public databases often suffer from incomplete metadata and the scarcity of functional assay data. Databases need to observe good standards for curating and maintaining the databases. The fields and vocabularies of metadata should be strictly controlled for efficient filtering and labeling. Moreover, the data should be processed through a unified pipeline, automated QCs, and active auditing to alleviate the burden of complex standardization or normalization among the data generated by different pipelines. For example, the ENCODE consortium operates the Data Coordination Center to define standard metadata fields, develop uniform data processing pipelines, and perform extensive data quality audits. Similarly, the HCA is developing standard operating procedures (SOPs) and standardized QCs to coordinate the scRNA-seq data submitted by the research community. The efforts made by the ENCODE consortium and the HCA to maintain the quality of data and metadata provide notable examples of how to improve the utility of public RB databases for DL. By adopting standardized and automated QC measures, developing a unified processing framework, and administering the metadata format and vocabularies, public databases will continue to function as a solid foundation for DL-powered RB research.

### Independent benchmarks

Standardized and unbiased benchmarking is a fundamental component of DL research, but this practice is uncommon in RB. Standard benchmark datasets are essential for quantifying the contribution of a new architecture, algorithm, or training strategy since study-specific benchmarks can often contain unintentional bias favoring their model. Multiple standard benchmark datasets are widely used in conventional fields of DL. For example, ImageNet^226^ and MS-COCO^227^ are widely used in computer vision, while SquAD^228^ and GLUE^229^ are commonly used in natural language processing. These benchmarks have enabled recent advances in DL by providing the means of fair and straightforward comparisons between models, allowing researchers to systematically evaluate the contribution of each novel architectural component and training technique. However, such standard benchmark datasets are not readily available in RB.

Individual studies have compared different DL models in RB using uniform criteria. These studies include benchmarks for nanopore sequencing basecalling^230^, RNA modification detection^160^, RBP binding site prediction^128,231^, miRNA–disease association prediction^232^, and gene expression prediction^233,234^. Notably, Sasse et al. and Huang et al. both benchmarked DL models for gene expression prediction and highlighted the generalizability issue. Enformer, Basenji2, ExPecto, and Xpresso all performed suboptimally for gene expression prediction in vivo. The authors also analyzed Enformer and showed that the model excessively relied on certain SNVs for expression level prediction. Also, Khan et al. performed an independent reevaluation of scBERT. The study showed that scBERT generalizes well to new independent datasets, but its performance is sensitive to class imbalance^235^.

These benchmark studies captured the strengths and weaknesses of the models that were not noted in the original studies, highlighting the importance of benchmarking. However, the lack of standard benchmark datasets prevents unbiased benchmarking from being adopted as a common practice in RB. Therefore, collective efforts should be directed toward developing standard benchmark datasets for various domains of RB.

### Computational efficiency

One evident trend in DL is the exponential growth of the model size, which improved the performance and generalizability of the models^236^. However, larger models require high-end hardware^237^. The limited affordability of this hardware impedes the dissemination of DL in biology, especially in academic laboratories^1^. Moreover, larger models require longer computation times. This limitation is problematic for time-critical field applications of biological DL, such as intraoperative^238^ or intensive care unit (ICU)^239^ patient diagnosis. Therefore, to reduce hardware requirements and computational time, improving the efficiency of RB DL models is crucial.

Several DL models and algorithms have been developed to allow the training and inference of DL models in resource-limited environments such as mobile and embedded systems^240^. The most common model-agonistic strategy is to utilize low-precision data types to reduce memory usage and increase throughput. Mixed precision is a widely adopted technique following this strategy. This technique uses high precision for updating the master model and low precision for gradient calculation during training^241^. Similarly, converting the model weight from floating point numbers to integers, a technique termed quantization, is an actively investigated avenue in DL for efficient inference^242^. Other ways to prepare the model for efficient inference include pruning, which is a discarding of unnecessary parameters to reduce the model size without impacting the performance^243^.

In addition to model-agonistic methods, various efforts have focused on designing lighter model architectures while maintaining the model performance. Some notable examples include MobileNet^244^, SqueezeNet^245^, ShuffleNet^246^, and EfficientNet^236^. These CNN models utilize various techniques aimed at capturing local features using fewer parameters. Researchers can use these techniques to increase the efficiency of biological DL models. For example, LegNet utilizes an EfficientNetV2-inspired^247^ architecture to predict gene expression from genomic sequences by capturing short regulatory elements^248^.

Recently, transformer-based models have dominated multiple fields of DL research. Therefore, the excessive computational burden has become more pronounced. As the input size grows linearly, the size of the transformer model grows quadratically. Therefore, it is difficult to use the transformer architecture for long inputs. To address this issue, multiple approximation algorithms, such as the linear transformer (249), Performer (250), and Linformer (251), have been proposed to scale transformers linearly with respect to the input size. Although these algorithms are not without drawbacks, including numerical instability and limited inputs, they can facilitate the adoption of the transformer architecture and its robust context-learning capability for RNA research. For example, scBERT utilizes the Performer architecture for cell type annotation from scRNA-seq data^44^. This design enables scBERT to receive a long input of 16,000 genes, which is significantly longer than what most language models can receive. In summary, leveraging architectures, algorithms, and techniques proven to improve the efficiency of model training and inference can facilitate the application of DL in RB, particularly in resource-limited settings such as clinical and field biology.

### Multimodality and structure

Humans can integrate diverse modes of information, such as audio, text, and vision, to produce a comprehensive understanding of the context. Recently, achieving such multimodality has emerged as a core task in DL, as it would improve the utility of models^249^. In RB, DL models can learn about biological processes and regulatory networks by integrating multiple biological modalities, including structural, evolutionary, genomic, epigenomic, transcriptomic, and proteomic data. There have been recent endeavors to apply multimodality to RB, including the studies reviewed in the previous section, such as CoraL, MOGONET, HE2RNA, DeepBind, Enformer, Aptardi, and PrismNet. For another example, Ashuach et al. developed a deep generative model, MultiVI, to integrate scATAC-seq, scRNA-seq, and surface protein expression data to generate a multiomics representation of a cell that can be used for data imputation^250^.

Structural data are an important modality that can be incorporated with transcriptomic data since they are a determinant of various processes involving RNA^251–253^. These data can be leveraged for various DL tasks, including predictions of RNA‒RNA interactions, gene expression, RNA modification, and splicing. The successful utilization of structural data for RBP binding prediction demonstrates their utility. However, the scarcity of experimentally determined RNA structures often impedes the incorporation of RNA structures as a DL feature. Multiple studies have used computational predictions of RNA structures as inputs to overcome this limitation^99,102,127,174^, even though the structure predictions have not reached the accuracy of the experiments. DL can improve the accuracy of RNA structure predictions, improving their utility as input features (Table 8 and Supplementary Table 7). ARES utilizes geometric DL to score RNA tertiary structure candidates^90^, SPOT-RNA and MXfold2 utilize CNNs to predict secondary structure^254,255^, trRosettaRNA utilizes transformer to predict tertiary structure^256^, and RoseTTAFoldNA utilizes transformer to predict the 3D structure of protein–nucleic acid complexes^179^. With the increasing performance of the deep RNA structure prediction models, it will be possible to include model-predicted RNA structures as input features for various RB tasks. The integration of diverse modalities, including RNA structures, is a prominent direction of DL research in RB that will allow a comprehensive and multiomics-level understanding of the transcriptome.

**Table 8:** Other DL models for RB.

Name	Input features	Task	Architecture	Paradigm	Ref.
SPOT-RNA	RNA sequence (one-hot)	RNA secondary structure prediction	CNN (ResNet)-BiLSTM hybrid	Supervised	^255^
DESC	Single-cell gene expression matrix	Cell clustering	Stacked autoencoder	Unsupervised	^41^
MARS	Single-cell gene expression matrix	Cell type annotation and novel cell type discovery	MLP	Meta-learning, Unsupervised	^58^
ARES	3D coordinates and chemical element types of atoms in RNA	RNA tertiary structure candidate scoring	Specialized architecture derived from tensor field network	Supervised	^90^
MXfold2	RNA sequence (embedding for each nucleotide)	RNA secondary structure prediction	CNN (ResNet)-BiLSTM hybrid	Supervised	^254^
scNym	Single-cell gene expression matrix	Cell type annotation	MLP with DANN	Semi-supervised	^55^
DNABERT	DNA sequence (embedding from k-mer tokens)	Promoter, splice site, and TF binding site prediction	Transformer	Self-supervised (masked contiguous token)	^271^
scBERT	Single-cell gene expression matrix	Cell type annotation and novel cell type discovery	Transformer	Self-supervised	^44^
GeMI	Unstructured metadata	Structured metadata generation	Transformer (GPT-2)	Supervised (Active learning)	^22^
BIONIC	Gene‒gene interaction (network adjacency matrix)	Chemical–genetic interaction prediction	GCN	Unsupervised and semi-supervised	^138^
RNA-FM	RNA sequence (embedding from nucleotide tokens)	RNA structure prediction, 5′ UTR ribosome loading prediction, and RNA‒protein interaction prediction	Transformer	Self-supervised	^274^
DNAGPT	DNA sequence (embedding from k-mer tokens)	Genomic signal (Poly(A) signal and TIS) recognition, mRNA abundance prediction, and artificial genome generation	Transformer	Self-supervised	^272^
trRosettaRNA	MSA and RNA secondary structure (probability matrix)	RNA tertiary structure prediction	Transformer	Supervised	^256^
Geneformer	Gene expression rank value encoding	Gene dosage sensitivity prediction, chromatin dynamics prediction, network dynamics prediction, and disease modeling	Transformer	Self-supervised	^45^
BigRNA	DNA sequence (one-hot)	Prediction of tissue-specific RNA expression, splicing, miRNA target sites, RBP specificity, and variant effects	Transformer	Self-supervised	^273^
MultIVI	Single-cell gene expression matrix and single-cell ATAC-seq matrix	scRNA-seq data imputation	MLP with DANN	Supervised	^250^

*GCN* graph convolutional network, *DANN* domain-adversarial neural network.

Models that are reviewed in the text but not included in Tables 1–7 are presented in the table.

### Interpretability

DL models have long been considered *black boxes*, a term symbolizing the difficulty of interpreting them. Interpretation is the process of understanding the reasoning behind the decisions of the models^257^. Unlike simple regression models, DL models cannot be thoroughly interpreted by inspecting their weights. Despite this difficulty, interpreting DL models is an essential step when applying DL to biological sciences, which pursues mechanistic explanations of biological processes. In addition, interpreting DL models can lead to the identification of novel biomarkers, drug candidates, and drug targets, which are important goals of medical biology^211^. Moreover, interpretation can contribute to identifying shortcomings and improving the performance of models.

Interpreting deep neural networks involves attributing the relevance of each feature or element to a task. The first approach for attribution is to perturb the input and then observe the change in the output. One example of this concept commonly used in computational biology is in silico saturation mutagenesis (ISM), in which each residue or nucleotide in a biological sequence is modified to every possible alternative, and a model quantifies the effect of the variants. For example, ISM was utilized with ExPecto to profile the effects of promoter-proximal variants on gene expression^191^. SHAP is another perturbation-based method that approximates results from all possible combinations of input elements to infer their individual contributions^258^. Janssens et al. and Almeida et al. both developed DL models to predict enhancer activity and interpreted the models with SHAP to assess nucleotide-wide contributions^259,260^.

The other major class of attribution methods is the gradient-based approach, which approximates the element- and feature-wise attributions for each given example by propagating the gradient. Intuitively, the gradient indicates the change in prediction that would result from a change in the input element. Popular methods of this type include saliency map^261^, gradient * input^262^, integrated gradients^263^, layerwise relevance propagation^264^, and DeepLIFT^265^. Multiple DL studies in RB have adopted gradient-based attribution methods to draw biological hypotheses from the models. For example, MultiRM utilizes integrated gradients to calculate the contribution of motifs in RNA modification prediction^76^, SpliceRover utilizes DeepLIFT to calculate nucleotide-wise importance in splicing^185^, and Enformer utilizes gradient * input to show that the model captures the importance of enhancer sequences in gene expression^132^. Notably, four attribution methods, gradient * input, integrated gradients, DeepLIFT, and layerwise relevance propagation, were tested using Xpresso, and it was found that all methods yielded similar results in terms of capturing the role of promoter regions in gene expression^192^. In addition, for transformer-based models, the weights of the attention matrices are often directly interpreted to identify the important input positions. This technique was used in MultiRM to identify the nucleotides involved in RNA modification deposition^76^ and in scBERT to identify core genes for cell type annotation^44^. As described above, multiple studies have successfully applied interpretation techniques to RB models to produce biological hypotheses. Nevertheless, caution should be taken when drawing scientific hypotheses from model interpretations. Current interpretation techniques only provide limited information, such as the contribution of each input, instead of demonstrating the systematic knowledge learned by the model^266^. To overcome this limitation, researchers can utilize biological domain knowledge to integrate interpretation results and discover novel functional and regulatory mechanisms of the transcriptome.

### Foundation models

Developing a system that can solve complex problems based on the knowledge established from large-scale data has been a major goal in DL. In this regard, models with billions of parameters have been trained on billions or trillions of data points. These models, termed the foundation models, have achieved unparalleled performance in a broad range of tasks without extensive task-specific training^267^. For instance, GPT-4 can describe input images in natural language and has excelled at AP and bar exams^268,269^. Foundation models are trained on large unlabeled datasets with self-supervised tasks. Representative tasks include causal language modeling, in which the model predicts the next word from the previous words^270^, and masked language modeling, in which the model regenerates masked-out portions of the original text^43^. Motivated by the success of foundation models in natural language processing, there have been attempts to develop foundation models for genomics and RB. Theoretically, a foundation model that has learned the language of biological sequences such as the genome, transcriptome, and proteome can systematically apply generalized knowledge to predict broad biological processes, including gene expression regulation, gene‒gene interactions, variant effects, and disease susceptibility.

There have been multiple efforts to develop foundation models for RB and genomics (Table 8 and Supplementary Table 7). DNABERT is one of the earliest attempts to construct such a model^271^. This model was pre-trained on the human genome sequence with a masked language modeling task, where DNA k-mers were regarded as words^43^. DNABERT exhibits a robust performance in predicting promoters, splice sites, and TF binding sites after fine-tuning with a limited amount of labeled data. The success of DNABERT supports the principle that modeling the genetic language can solve various downstream genomics tasks. While DNABERT was trained on the genomic sequence, Geneformer was trained on the human single-cell transcriptome to learn tissue-specific knowledge of gene expression regulation networks^45^. The model was trained on gene expression ranks extracted from 29.9 million human single-cell RNA-seq data. A masked language modeling task, where genes are regarded as words, was used for pre-training. The trained model showed robust performance in gene dosage sensitivity, chromatin dynamics, and network dynamics tasks. Furthermore, the model identified candidate therapeutic targets for cardiomyopathy by modeling the pathology at the gene network level, suggesting the potential value of RNA foundation models in medicine.

While DNABERT and Geneformer were pre-trained on a single type of data or task, DNAGPT was pre-trained on multiple types of data and tasks. DNAGPT was pre-trained on mammalian genomes using multiple tasks including causal language modeling regarding DNA k-mers as words, binary classification of DNA sequence order, and numerical regression of guanine-cytosine (GC) content^272^. The model was able to predict gene expression and recognize functional regions such as poly(A) signals and translation initiation sites. The authors also showed that DNAGPT can generate artificial human genomes. However, sophisticated methods to measure the quality of the generated genome need to be devised to fully investigate the potential of artificial genome generation. BigRNA combined the prediction of multiple models to improve the generalizability, the technique called ensemble. This model was pre-trained to predict gene expression from genomic sequences and was fine-tuned to predict RBP and miRNA binding^273^. In contrast to other foundation models that are primarily pre-trained on DNA sequences, RNA-FM was pre-trained on noncoding RNA sequences using masked language modeling^274^. Notably, while structural data were not used for the pre-training, RNA-FM was able to predict RNA structure in terms of the secondary structure and 3D distances, in addition to functional features, including RBP and ribosome binding.

While multiple studies have made significant contributions toward developing foundation models in genomics and RB, the performance of the model and the diversity of the downstream tasks need to be improved. Importantly, foundation models should exhibit emergent properties and solve novel biological problems that have not been answered by task-specific models. By integrating recent advances in network biology and systems biology^275,276^, it will be possible to develop foundation models that learn comprehensive knowledge of the biological language.

### AI safety

As the capabilities of DL models expand in an accelerating phase, concerns regarding their safety are rising. One of the major concerns is the racial and sex biases of the models, which often manifest in the form of representational bias and performance disparities^267^. For instance, if a dataset used to train a DL model underrepresents a specific subpopulation, the model will struggle to make accurate inferences about that group. This problem is not new in biomedical research, where the inclusion of women and minority groups has been recognized as an essential goal to prevent racial and sex bias in research outcomes and improve the generalizability of the research outcome^277^. Since numerous DL models for RB are trained with datasets constructed using public databases, it is vital to maintain the racial, ethnic, and sexual diversity of ex vivo data in the databases. However, many public databases exhibit insufficient diversity^278^. For example, the vast majority of the GTEx v8 data are derived from individuals of European descent (85.3%), significantly underrepresenting the Asian population (1.4%), American Indian or Alaska Native populations (0.2%), and individuals identifying as Hispanic or Latino (1.9%)^279^. Moreover, the database underrepresents females (33.5%), introducing the source of potential sex bias. TCGA, another major source of ex vivo RNA-seq data, also suffers from the overrepresentation of White people (77%) and underrepresentation of Asian (3%) and Hispanic (3%) peoples^280^. These imbalances in public databases can facilitate the model to learn social biases against marginalized and underrepresented populations.

The most straightforward measure to mitigate social biases while training DL models is to resample data to achieve balanced racial and sex distributions^281^. However, adopting this strategy is difficult since it severely reduces the number of training data points, which is already scarce in most fields of RB. Another strategy is to utilize data augmentation techniques to oversample underrepresented populations. However, this approach is uncommon because it can degrade the model performance and introduce additional biases^282^. Therefore, the ideal solution would be to collect additional biological data from the underrepresented populations to counteract the existing bias. The All of Us Research Program of the NIH is one example of such efforts^283^. This program aims to collect genomic and health data from more than one million participants, focusing on traditionally underrepresented populations. Although this program does not currently collect transcriptomic data, similar efforts can be made in RB to enhance the social impact of biological DL studies.

Protecting the privacy and security of human-derived biological data is another crucial task in AI safety. This issue is especially emphasized in RB, considering the extensive amount of sensitive biological and clinical information that can be mined from transcriptomic data^284^. Indeed, the most straightforward way to prevent privacy breaches is to utilize only data that have been consented for public release, similar to the practice of the ENCODE and GEO databases^285^. However, a substantial portion of clinical data does not come with such consent, which leads to the demand for privacy-preserving machine learning^286^. It is a common practice to de-identify biomedical data before providing them for DL studies^287^. However, extensive de-identification can remove valuable information from the data, and there is a risk of re-identification using existing techniques^288^. Employing fully homomorphic encryption can overcome these limitations and protect privacy^289^. Fully homomorphic encryption allows DL models to be trained directly with encrypted data because its defining property is that arbitrary operation on encrypted data followed by decryption should yield results identical to those of operations on decrypted data^290^. Therefore, fully homomorphic encryption eliminates the need for extensive de-identification and allows the complete utilization of sensitive transcriptomic data for deep learning-powered RB research without privacy risks.

Another important technique to protect privacy in DL is federated training. Federated learning is a method that trains multiple independent DL agents locally and subsequently aggregates the weight updates on a central agent^291^. Using federated learning, multiple institutions can collaborate without sharing sensitive transcriptome data outside the institution^292,293^. It is even possible to eliminate the necessity of a central server using blockchain, further reducing the risk of privacy breaches^294^. In summary, adopting appropriate techniques can improve the safety of DL models in RB, expanding the range of training data and ensuring fair distribution of the potential benefits of RNA DL research across society.

## Prospects and promises of deep learning in RNA biology

The exponential growth of big data in RB has opened new opportunities for employing DL in RNA research. DL has assisted in numerous discoveries and progresses in the study of RNA-binding proteins, ncRNAs, epitranscriptome, pre-mRNA processing, and gene expression. Moreover, DL models have leveraged RB data for medical applications, including diagnosis and drug development. Researchers have designed architectures to utilize biological data more effectively and have employed algorithms and techniques to counteract the data limitations. These efforts have resulted in the development of novel tools to assist and streamline RNA research.

Numerous DL studies reviewed in this article were made possible by public databases providing high-throughput experimental data. This fact highlights the central importance of public databases for employing DL in RB. The construction of better DL models for RB requires better datasets for training and benchmarking. Public databases need to focus on metadata integrity and QC, which can be achieved by developing unified analysis pipelines for each functional genomics assay and sequencing platform. Moreover, efforts should be directed toward compiling unbiased large-scale public datasets that can be used as standards for benchmarking RB models, which are central to engineering DL models.

Reflecting on the inspirational success of foundation models in understanding natural languages, it seems rational to expect that a DL model can be trained to decipher the genomic and transcriptomic language. Developing a foundation model for RB requires appropriate adaptations of self-supervised learning and multimodality paradigms for RB. Specifically, large-scale transcriptomic corpora, pre-training tasks for biological languages, downstream tasks for functional genomics, and standardized benchmarks have to be developed. While there have been various pioneering works in this direction, we believe that the paradigm-shifting potential of DL has yet to be fully realized in RB.

In summary, DL has shown immense potential in RB, and we expect it to play a significant role in unraveling the mysteries of RB in the future. Recently, DL has demonstrated its ability to guide the conception of novel scientific hypotheses^295^. With the ongoing technological advancements in functional genomics and the increasing availability of big data, researchers need to continue exploring the potential of DL in RB to accelerate and facilitate scientific discoveries.

### Supplementary information

Supplementary Information for Big Data and Deep Learning for RNA Biology
