# Supplementary material for: Big data and deep learning for RNA biology
Source: Exp Mol Med. 2024 Jun 14;56(6):1293–321. doi: 10.1038/s12276-024-01243-w (PMC11263376; doi:10.1038/s12276-024-01243-w)
Supplement: Supplementary file 1 — Supplementary Information for Big Data and Deep Learning for RNA Biology [file 12276_2024_1243_MOESM1_ESM.pdf]

Supplementary Information for
Big Data and Deep Learning for RNA Biology

Hyeonseo Hwang[1,#], Hyeonseong Jeon[2,3,#], Nagyeong Yeo[1,#], & Daehyun Baek[1,2,3,*]

[1] School of Biological Sciences, Seoul National University, Seoul, 08826, Republic of Korea

[2] Interdisciplinary Program in Bioinformatics, Seoul National University, Seoul, 08826, Republic of Korea

[3] Genome4me, Inc., Seoul, 08826, Republic of Korea

[#] These authors contributed equally: Hyeonseo Hwang, Hyeonseong Jeon, and Nagyeong Yeo.

[*] Correspondence should be addressed to Daehyun Baek (baek@snu.ac.kr).

This PDF file includes:

Supplementary Tables 1-7

Supplementary References

NAME	TRAINING DATASET	EVALUATION DATASET
DeepMirTar	miRNA-target interaction data by mirMark [1] and CLASH [2], miRNA sequences from miRBase [3], 3′ UTR sequence from UCSC Genome Browser	†, published iRNA-target interaction data by PAR-CLIP [4,5]
LncADeep	Annotation from RefSeq and GENCODE, lncRNA-protein interaction from NPInter [6]	†
mRNN	Annotation from GENCODE	†, Annotation from GENCODE including mRNAs with short ORFs and lncRNAs with long untranslated ORFs
Aoki *et al*.	ncRNA sequences from Ensembl and GtRNAdb [7]	†
TargetScan 8	In-house AGO-RBNS and miRNA transfection followed by RNA-seq	In-house miRNA transfection followed by RNA-seq
dnnMiRPre	miRNA precursor sequence from miRBase, non-miRNA hairpin sequence from RefSeq	†
circDeep	circRNA sequence from circRNADb, annotation from GENCODE, conservation score from UCSC genome browser	†
RNAsamba	Annotation from GENCODE and RefSeq (CPC2 [8], FEELnc [9], mRNN datasets [10])	†
SG-LSTM-FRAME	PPI data from BioGRID [11], gene sequence acquired via biomaRT, miRNA sequence from miRBase, miRNA-gene interaction from miRTarBase [12]	miRNA-gene interaction from miRTarBase
DeepLncPro	Eukaryotic Promoter Database [13]	†
Finetuned DNABERT	circRNA-miRNA interaction data from the KDGCMI I dataset [14]	†
DiMo	miRNA sequences from TargetScan Database [15] and starBase [16]	†
GM-lncLoc	lncLocator [17] and iLoc-lncRNA [18] datasets from the RNALocate database	†

Supplementary Table 1. Datasets used for training DL models for ncRNA biology.

† = Identical to the left cell.

NAME	TRAINING DATASET	EVALUATION DATASET
deepnet-rbp	23 PAR-CLIP and iCLIP datasets from DoRiNA [19], 1 published HITS-CLIP dataset [20]	†
DeepBind	Published RNAcompete data [21]	†
iDeepS	CLIP data from DoRiNA [19] and iCount	†
iDeepE	CLIP data from GraphProt [22] and RNAcommender [23] datasets	†
pyssster	CLIP data from ssHMM dataset [24]	†
DLPRB	Published RNAcompete data [21], published icSHAPE data [25]	†, eCLIP data from ENCODE, CLIP data from GraphProt dataset
Avsec *et al.*	eCLIP data from ENCODE, annotation from GENCODE	†, Curated CLIP benchmark dataset [26] [27]
RPI-Net	CLIP data from GraphProt dataset	†
DeepCLIP	CLIP data from GraphProt dataset	eCLIP data from POSTAR [28] and ENCODE
PrismNet	CLIP data from POSTAR, eCLIP data from ENCODE [29], and in-house icSHAPE data	†
RoseTTAFoldNA	Protein, RNA, and protein-nucleic acid complex structures from PDB	†

Supplementary Table 2. Datasets used for training DL models for RNA-protein interactions. The use of † is the same as in Supplementary Table 1.

NAME	TRAINING DATASET	EVALUATION DATASET
Deep-2'-O-Me	Published 2'-O-Me site data by Nm-seq [30]	†
pysster	A-to-I editing site data from REDIportal [31]	†
iPseU-CNN	Pseudouridine site data from iRNA-PseU dataset [32]	†
Gene2Vec	RNA sequence from Ensembl, published m6A site data [33,34]	†
MultiRM	20 published epitranscriptomic profiles	†
Dinopore	in-house Nanopore DRS data, in-house ADAR1 KO data, A-to-I editing site data from REDIportal, SNP data from dbSNP [35]	†
iM6A	Published m6A-CLIP data [36-38] [39], annotation data from GENCODE	†, Published m6A-label-seq data [40], published MAZTER-seq data [41], published m6ACE-seq data [42]
m6Anet	Published Nanopore DRS data [43-46], published m6ACE-seq data [42,43]	†, published Nanopore DRS data [44], published miCLIP data [38]
Deepm5C	m5C site data from m6A-Atlas [47]	†

Supplementary Table 3. Datasets used for training DL models for epitranscriptomics.

The use of † is the same as in Supplementary Table 1. KO = knockout, SNP = single-nucleotide polymorphism, DRS = direct RNA sequencing.

NAME	TRAINING DATASET	EVALUATION DATASET
Leung *et al*. (2014)	Published RNA-Seq data [48]	†, published RNA-Seq data [49]
Xiong *et al*.	RNA-seq data from the Illumina Human BodyMap 2.0 project	†; published RNA-seq data [48]; RNAcompete data [21]; RNA-seq data [50]; SNV data from dbSNP, HGMD [51], InSIGHT [52], and ANNOVAR [53]; in-house whole-genome sequencing data;
Jha *et al*.	Published RNA-Seq data [48,54], 15 CLIP datasets from SRA and ArrayExpress, 7 RNA-Seq datasets from GEO	†
Leung *et al*. (2018)	Published polyA-Seq data [55], published 3′-seq data [56], 3′-UTR annotation from GENCODE, APADB [57], and PolyA_DB 2 [58]	†, variant data from ClinVar [59]
DeepPolyA	Poly(A) site data from a published dataset [60], negative sequences from the Arabidopsis Information Resources database [61]	†, motif data from the JASPAR database [62]
SpliceRover	Published splicing data [63-66]	†
DeepIsoFun	Isoform sequences from RefSeq, 1735 RNA-Seq experiments from SRA, functional annotation from UniProt GO database	†
COSSMO	RNA-Seq data from GTEx and annotation from GENCODE	†
Avsec et al.	Published branchpoint annotation [67] and genome annotation from GENCODE	†
DeeReCT-PolyA	Poly(A) signal annotation from GENCODE and published datasets (dragon-human [68], omni-human [69])	†
DeepGSR	Poly(A) signal and TIS data extracted from cDNA data retrieved from NCBI, UCSC genome browser, Ensembl, Mammalian Gene Collection [70], FlyBase [71], and Ensembl	†
DeepPASTA	Published PolyA-Seq data [55]	†, Poly(A) site data from a published dataset [72]
APARENT	MPRA for APA (in-house)	†, variant data from ClinVar, APA data from APADB and Leslie APA Atlas [56], RNA-Seq data from GEUVADIS [73]
DARTS	RNA-seq data from ENCODE and Roadmap Epigenomics	†, published RNA-seq data [74-76], in-house RASL-seq data
SpliceAI	Annotation from GENCODE	†; RNA-seq data from GTEx and in-house experiments; splicing variant data from ExAC [77] and gnomAD; *de novo* mutation data from Deciphering Developmental Disorders [78], Simons Simplex Collection [79], and Consortium [80]
MMSplice	Annotation from GENCODE, RNA-seq data from GTEx, published alternative splicing data by MPRA, and [81] Vex-seq [82]	†, splicing variant data from ClinVar and ExAC, published splicing efficiency data by MaPSy [83]
SANPolyA	Poly(A) signal annotation from GENCODE and published datasets (dragon-human, omni-human, GSR-human [84], BL [85], and SP [85])	†
Aptardi	RNA-seq data from Human Brain Reference [86], published human PolyA-seq data [55],	†, RNA-seq data from Universal Human Reference [87], published rat RNA-seq data [88], published CFIm25

		KO human RNA-Seq data [89], published Mouse RNA-Seq data [90], published mouse and rat PolyA-seq data [55]
DMIL-IsoFun	Maize genome assembly from MaizeGDB [91], 10 RNA-seq datasets from SRA	†
Pangolin	Published RNA-seq data [92], annotations from GENCODE and Ensembl	†, Splice variant data by MFASS from ExAC, published splicing efficiency data by MaPSy

Supplementary Table 4. Datasets used for training DL models for pre-mRNA processing.

The use of † is the same as in Supplementary Table 1. SNP = single nucleotide polymorphism, HGMD = Human Gene Mutation Database, GO = gene ontology, cDNA = complementary DNA, RASL-seq = RNA-mediated oligonucleotide annealing, selection, and ligation with next-generation sequencing, ExAC = Exome Aggregation Consortium, gnomAD = Genome Aggregation Database, MaPSy = massively parallel splicing assay, KO = knockout, MFASS = multiplexed functional assay of splicing using Sort-seq.

NAME	TRAINING DATASET	EVALUATION DATASET
DeepChrome	Histone modification data from Roadmap Epigenomics	†
D-GEX	Microarray expression data from GEO (training), RNA-Seq expression data from The 1000 Genomes (validation)	RNA-Seq expression data from GTEx
Cuperus *et al.*	Liquid-based growth assay of 5′UTRs (in-house)	†, HIS3 assay (in-house)
ExPecto	Tissue expression profiles from GTEx, ENCODE, and Roadmap epigenomics; CAGE reads from FANTOM5	†, GTEx v6 eQTLs, the 1000 Genomes phase 3, and GWAS Catalog data[93], regulatory mutation data from HGMD
DeepDiff	Histone modification data from Roadmap Epigenomics	†
Basenji	DNase-seq and histone modification ChIP-seq data from ENCODE and Roadmap, CAGE data from FANTOM5	†, eQTL statistics from GTEx, SNPs from GWAS Catalog database
Optimus 5-Prime	5' UTR MPRA data (in-house)	†, human 5' UTR transcripts from Ensembl, sequence variants from ClinVar
DeepExpression	Genomic data from Ensembl, *S. cerevisiae* genomic data from Saccharomyces Genome Database[94,95], published transcript and ORF boundaries of *S. cerevisiae* [96,97], *E. coli* genomic data from RegulonDB[98], RNA-seq data from Digital Expression Explorer V2[99]	†, Published gene expression dataset in yeast [100] [101], published RNA-Seq experiments from 6 different organisms, TF binding sites data from JASPAR[102] and Yeastract database[103]
DEcode	TPM from human tissues and whole-genome sequences from GTEx, binding sites of RBPs from POSTAR2[28], binding sites of TFs from GTRD[104], binding sites of miRNAs from TargetScan7[105]	†, LOF mutation data from ExAC [106], disease gene data from Online Mendelian Inheritance in Man, murine KO experimental data from Mouse Genome Informatics [107] and the International Mouse Phenotyping Consortium [108], CRISPR screening data from DepMap[109], KEGG pathway gene sets from MSigDB [110]
Basenji2	DNase-seq, ChIP-seq, CAGE, and ATAC-seq data from ENCODE, FANTOM, and GEO, including published ATAC-seq data[111]	†, eQTL statistics from GTEx, pathogenic non-coding variants from ClinVar and HGMD, published data of fine-mapped GWAS variants [112] [113], WGS data from Simons Simplex Collection
Xpresso	RNA-seq data from Roadmap epigenomics, CAGE annotations from FANTOM5, TSS annotations from Ensembl, human-to-mouse orthologues data from Ensembl	†, published mRNA half-life measurements [114] [115], published miRNA data from mESCs [116], published SuRE MPRA data [117]
Enformer	Basenji2 [118], CAGI5 competition [119]	†, published CRISPRi data [120] [121], H3K27ac ChIP-seq data and DNase-seq data from ENCODE, gene expression summary statistics for 48 tissues from GTEx, published TAD boundary data [122], published fine-mapped eQTLs [123]
TFCNN	RNA-Seq and ChIP-seq data from ENCODE	†
HiCoEx	RNA-seq data from GTEx and TCGA, published Hi-C data from 12 types of tissues and cell lines, and published RNA-seq data [124]	†
TISnet	Ribo-seq, RNA-seq, and SHAPE-MaP (in-house)	†

GEARS	Published perturbation data [125]	†, 7 different published perturbation datasets
LegNet	DREAM2022 challenge, Published Yeast GPRA [126]	†

Supplementary Table 5. Datasets used for training DL models for gene expression.

The use of † is the same as in Supplementary Table 1. HGMD = Human Gene Mutation Database, TPM = Transcripts per million, GTRD = Gene Transcription Regulation Database, LOF = loss-of-function, KO = knockout, TSS = transcription start site, mESC = mouse embryonic stem cell, SuRE = Survey of Regulatory Elements, TAD = topologically associating domain, GPRA = gigantic parallel reporter assay.

NAME	TRAINING DATASET	EVALUATION DATASET
Young *et al*.	Transcriptional data from TCGA	†
DeepCRISPR	4 published on-target datasets; 7 published off-target datasets; epigenetic data generated by ChIP-seq, DNase-seq, and RRBS assay from ENCODE	†
DeepSEA (extended)	Histone marks, TFs, and DNase I profiles from ENCODE and Roadmap Epigenomics; CLIP and eCLIP data from ENCODE; published CLIP data; Non-coding mutations from HGMD; Rare variants from 1000 Genomes	†, ASD WGS data from SFARI base, gene expression data of 53 tissues from GTEx
CUP-AI-Dx	RNA-seq data from TCGA and ICGC	†, RNA-seq data from FFPE clinical samples (in-house), published microarray data [127,128]
HE2RNA	Whole-slide images and corresponding RNA-seq data from TCGA	†, published colorectal cancer histology slides [129], liver hepatocellular carcinoma sample slides [130], published epithelium slides[131]
Qiu *et al*.	RNA-seq data from TCGA	†; published non-small cell lung cancer cohort data [132]; high-risk and low-risk gene sets from KEGG, The Reactome Pathway Knowledgebase[133], and WikiPathways[134]
IMX-BVN-1	Published transcriptional data from 18 different studies from GEO and ArrayExpress	†, NanoString data of 163 patients in the Stanford ICU Biobank
CRISPRon	in-house and published [135] gRNA activity data	†
MOGONET	Multi-omics-data of Alzheimer's Disease patients from ROSMAP [136,137]; Multi-omics-data of low-grade glioma grade, kidney cancer, and breast invasive carcinoma from TCGA; breast cancer subtype information from PAM50 [138,139]	†
BE-DICT	DNA sequencing data of base editing outcomes (in-house), disease-associated SNPs from ClinVar	†, published base editing data [140,141]
Gao *et al*.	RNA-seq data of BPN-15477 treated cells (in-house)	†
Wayment-Steele *et al*.	Published PERSIST-seq data [142]	†, in-house in-line-seq data
scDEAL	Bulk RNA-seq drug response data from GDSC [143] and DepMap [144]	†, 6 scRNA-seq drug response datasets retrieved from GEO
TIGER	In-house gRNA activity data	†, published gRNA activity data [145], CRISPR screening data from DepMap [109],
PRIDICT	Prime editing efficiency data (in-house), target sequences of pathogenic variants from ClinVar	†, Published prime editing data [146-148]
CoraL	Functional peptide dataset from BPD [149], cancer-associated ncPEPs data, and short open reading frame data from SPENCER [150]	†
OTTER	RNA-seq data of tumor samples from UCSC Treehouse Childhood Cancer Initiative Compendium [151] and St. Jude Children's Hospital Pediatric Cancer Genome Project	†, single-cell RNA-seq data(in-house)

	[152], RNA-seq data of normal tissue samples from GTEx	
shRNAI+	Gene annotation data from GENCODE, published Drosha processing data [153], 3 published shRNA processing and potency datasets.	†, 3 published shRNA processing and potency datasets, 5 published shRNA screening datasets

Supplementary Table 6. Datasets used for training DL models for medical applications of RB.

The use of † is the same as in Supplementary Table 1. RRBS = reduced representation bisulfite sequencing, HGMD = Human Gene Mutation Database, WGS = whole-genome sequencing, SFARI = Simons Foundation Autism Research Initiative, SNP = single-nucleotide polymorphism, GDSC = Genomics of Drug Sensitivity in Cancer, BPD = bioactive peptide discovery.

NAME	(PRE-) TRAINING DATASET	EVALUATION / FINE-TUNING DATASET
SPOT-RNA	RNA sequences and secondary structures from bpRNA [154] and PDB	†
DESC	6 published scRNA-seq datasets retrieved from GEO and ArrayExpress	†
MARS	scRNA-seq data from Tabula Muris [155] and Tabula Muris Senis [156]	†, 7 additional published scRNA-seq datasets
ARES	RNA structures from FARNA [157] and FARFAR2 [158]	†, RNA structures from RNA-Puzzles [159]
MXfold2	6 RNA secondary structure datasets from publications and Rfam [160]	†
scNym	scRNA-seq data from Rat Aging Cell Atlas [161]	†
DNABERT	Human genome sequence	Human promoters from EPDnew [162]; TF ChIP-seq data from ENCODE and GEO [163,164]; Splice sites from SpliceFinder dataset [165]
scBERT	scRNA-seq data from PanglaoDB [166] and heart scRNA-seq dataset [167] from HCA	scRNA-seq data from HCA, Zheng68k dataset [168], and 7 other publications
GeMI	Metadata from Cistrome [169] and ENCODE	†, metadata from GEO
BIONIC	Protein-protein interaction network from BioGRID [11], gene expression profiles from SPELL database [170], and published genetic interaction profiles [171]	Functional annotations from IntAct [172], KEGG, and GO; published chemical-genetic screen data [173]
RNA-FM	ncRNA sequences from RNAcentral [174]	RNA secondary structure from 3 published datasets [154,175,176], a published RNA tertiary structure dataset [177], and a published 5'UTR MPRA dataset [178]
DNAGPT	Reference genomes from Ensembl	Poly(A) signal and TIS from DeepGSR dataset [84], SNPs from 1000 Genomes [179], CAGE data from FANTOM5
trRosettaRNA	MSA from NCBI nt database, Rfam, and RNAcentral; RNA structures from RNA-Puzzles, PDB, and bpRNA	†, RNA structure data from CASP15
Geneformer	Genecorpus-30M, which is a human single-cell transcriptome dataset compiled from 561 public sources, including GEO, SRA, and HCA	Heart scRNA-seq dataset [167] from HCA, published dosage-sensitive gene list [180], bivalent gene data from PanglaoDB and publication [181]
BigRNA	RNA-seq data from GTEx	eCLIP data from ENCODE, pathogenic variants from ClinVar, PolyA sites from PolyASite 2.0 [182], splicing variants from SPCards [183], benign variants from gnomAD, published splicing efficiency data by MaPSy
MultIVI	scRNA- and scATAC-seq data of 10X multiomic unsorted PBMC dataset	†, published single-cell multimodal datasets [184-187] retrieved from GEO

Supplementary Table 7. Datasets used for training Other DL models for RB.

The use of † is the same as in Supplementary Table 1. MSA = multiple sequence alignment, MaPSy = massively parallel splicing assay, PBMC = peripheral blood mononuclear cell.

Supplementary References

1 Menor, M., Ching, T., Zhu, X., Garmire, D. & Garmire, L. X. mirMark: a site-level and UTR-level classifier for miRNA target prediction. *Genome biology* **15**, 1-16 https://doi.org/10.1186/s13059-014-0500-5 (2014).

2 Helwak, A., Kudla, G., Dudnakova, T. & Tollervey, D. Mapping the Human miRNA Interactome by CLASH Reveals Frequent Noncanonical Binding. *Cell* **153**, 654-665 https://doi.org/https://doi.org/10.1016/j.cell.2013.03.043 (2013).

3 Kozomara, A., Birgaoanu, M. & Griffiths-Jones, S. miRBase: from microRNA sequences to function. *Nucleic Acids Research* **47**, D155-D162 https://doi.org/10.1093/nar/gky1141 (2019).

4 Hafner, M. *et al.* Transcriptome-wide Identification of RNA-Binding Protein and MicroRNA Target Sites by PAR-CLIP. *Cell* **141**, 129-141 https://doi.org/10.1016/j.cell.2010.03.009 (2010).

5 Hafner, M. *et al.* Transcriptome-wide identification of RNA-binding protein and microRNA target sites by PAR-CLIP. *Cell* **141**, 129-141 https://doi.org/10.1016/j.cell.2010.03.009 (2010).

6 Yuan, J. *et al.* NPInter v2.0: an updated database of ncRNA interactions. *Nucleic Acids Research* **42**, D104-D108 https://doi.org/10.1093/nar/gkt1057 (2013).

7 Chan, P. P. & Lowe, T. M. GtRNAdb: a database of transfer RNA genes detected in genomic sequence. *Nucleic acids research* **37**, D93-D97 https://doi.org/10.1093/nar/gkn787 (2009).

8 Kang, Y.-J. *et al.* CPC2: a fast and accurate coding potential calculator based on sequence intrinsic features. *Nucleic acids research* **45**, W12-W16 https://doi.org/10.1093/nar/gkx428 (2017).

9 Wucher, V. *et al.* FEELnc: a tool for long non-coding RNA annotation and its application to the dog transcriptome. *Nucleic acids research* **45**, e57-e57 https://doi.org/10.1093/nar/gkw1306 (2017).

10 Hill, S. T. *et al.* A deep recurrent neural network discovers complex biological rules to decipher RNA protein-coding potential. *Nucleic Acids Research* **46**, 8105-8113 https://doi.org/10.1093/nar/gky567 (2018).

11 Chatr-Aryamontri, A. *et al.* The BioGRID interaction database: 2013 update. *Nucleic acids research* **41**, D816-D823 https://doi.org/10.1093/nar/gks1158 (2012).

12 Chou, C.-H. *et al.* miRTarBase update 2018: a resource for experimentally validated microRNA-target interactions. *Nucleic acids research* **46**, D296-D302 https://doi.org/10.1093/nar/gkx1067 (2018).

13 Meylan, P., Dreos, R., Ambrosini, G., Groux, R. & Bucher, P. EPD in 2020: enhanced data visualization and extension to ncRNA promoters. *Nucleic Acids Research* **48**, D65-D69 https://doi.org/10.1093/nar/gkz1014 (2020).

14 Wang, X. F. *et al.* KGDCMI: A New Approach for Predicting circRNA–miRNA Interactions From Multi-Source Information Extraction and Deep Learning. *Frontiers in Genetics* **13** https://doi.org/10.3389/fgene.2022.958096 (2022).

15 Friedman, R. C., Farh, K. K., Burge, C. B. & Bartel, D. P. Most mammalian mRNAs are conserved targets of microRNAs. *Genome Res* **19**, 92-105 https://doi.org/10.1101/gr.082701.108 (2009).

16 Li, J.-H., Liu, S., Zhou, H., Qu, L.-H. & Yang, J.-H. starBase v2. 0: decoding miRNA-ceRNA, miRNA-ncRNA and protein–RNA interaction networks from large-scale CLIP-Seq data. *Nucleic acids research* **42**, D92-D97 https://doi.org/10.1093/nar/gkt1248 (2014).

17 Cao, Z., Pan, X., Yang, Y., Huang, Y. & Shen, H. B. The lncLocator: a subcellular localization predictor for long non-coding RNAs based on a stacked ensemble classifier. *Bioinformatics* **34**, 2185-2194 https://doi.org/10.1093/bioinformatics/bty085 (2018).

18 Su, Z.-D. *et al.* iLoc-lncRNA: predict the subcellular location of lncRNAs by incorporating octamer composition into general PseKNC. *Bioinformatics* **34**, 4196-4204 https://doi.org/10.1093/bioinformatics/bty508 (2018).
19 Anders, G. *et al.* doRiNA: a database of RNA interactions in post-transcriptional regulation. *Nucleic acids research* **40**, D180-D186 https://doi.org/10.1093/nar/gkr1007 (2012).
20 Xue, Y. *et al.* Genome-wide analysis of PTB-RNA interactions reveals a strategy used by the general splicing repressor to modulate exon inclusion or skipping. *Molecular cell* **36**, 996-1006 https://doi.org/10.1016/j.molcel.2009.12.003 (2009).
21 Ray, D. *et al.* A compendium of RNA-binding motifs for decoding gene regulation. *Nature* **499**, 172-177 https://doi.org/10.1038/nature12311 (2013).
22 Maticzka, D., Lange, S. J., Costa, F. & Backofen, R. GraphProt: modeling binding preferences of RNA-binding proteins. *Genome Biol* **15**, R17 https://doi.org/10.1186/gb-2014-15-1-r17 (2014).
23 Corrado, G., Tebaldi, T., Costa, F., Frasconi, P. & Passerini, A. RNAcommender: genome-wide recommendation of RNA-protein interactions. *Bioinformatics* **32**, 3627-3634 https://doi.org/10.1093/bioinformatics/btw517 (2016).
24 Heller, D., Krestel, R., Ohler, U., Vingron, M. & Marsico, A. ssHMM: extracting intuitive sequence-structure motifs from high-throughput RNA-binding protein data. *Nucleic Acids Res* **45**, 11004-11018 https://doi.org/10.1093/nar/gkx756 (2017).
25 Spitale, R. C. *et al.* Structural imprints in vivo decode RNA regulatory mechanisms. *Nature* **519**, 486-490 https://doi.org/10.1038/nature14263 (2015).
26 Pan, X. & Shen, H. B. RNA-protein binding motifs mining with a new hybrid deep learning based cross-domain knowledge integration approach. *BMC Bioinformatics* **18**, 136 https://doi.org/10.1186/s12859-017-1561-8 (2017).
27 Stražar, M., Žitnik, M., Zupan, B., Ule, J. & Curk, T. Orthogonal matrix factorization enables integrative analysis of multiple RNA binding proteins. *Bioinformatics* **32**, 1527-1535 https://doi.org/10.1093/bioinformatics/btw003 (2016).
28 Zhu, Y. *et al.* POSTAR2: deciphering the post-transcriptional regulatory logics. *Nucleic acids research* **47**, D203-D211 https://doi.org/10.1093/nar/gky830 (2019).
29 Van Nostrand, E. L. *et al.* Robust transcriptome-wide discovery of RNA-binding protein binding sites with enhanced CLIP (eCLIP). *Nature Methods* **13**, 508-514 https://doi.org/10.1038/nmeth.3810 (2016).
30 Dai, Q. *et al.* Nm-seq maps 2′-O-methylation sites in human mRNA with base precision. *Nature Methods* **14**, 695-698 https://doi.org/10.1038/nmeth.4294 (2017).
31 Picardi, E., D'Erchia, A. M., Lo Giudice, C. & Pesole, G. REDIportal: a comprehensive database of A-to-I RNA editing events in humans. *Nucleic Acids Res* **45**, D750-d757 https://doi.org/10.1093/nar/gkw767 (2017).
32 Chen, W., Tang, H., Ye, J., Lin, H. & Chou, K. C. iRNA-PseU: Identifying RNA pseudouridine sites. *Mol Ther Nucleic Acids* **5**, e332 https://doi.org/10.1038/mtna.2016.37 (2016).
33 Zhou, Y., Zeng, P., Li, Y. H., Zhang, Z. & Cui, Q. SRAMP: prediction of mammalian N6-methyladenosine (m6A) sites based on sequence-derived features. *Nucleic Acids Res* **44**, e91 https://doi.org/10.1093/nar/gkw104 (2016).
34 Xiang, S., Liu, K., Yan, Z., Zhang, Y. & Sun, Z. RNAMethPre: A Web Server for the Prediction and Query of mRNA m6A Sites. *PLoS One* **11**, e0162707 https://doi.org/10.1371/journal.pone.0162707 (2016).
35 Sherry, S. T. *et al.* dbSNP: the NCBI database of genetic variation. *Nucleic acids research* **29**, 308-311 https://doi.org/10.1093/nar/29.1.308 (2001).
36 Ke, S. *et al.* A majority of m6A residues are in the last exons, allowing the potential for 3' UTR regulation. *Genes Dev* **29**, 2037-2053 https://doi.org/10.1101/gad.269415.115 (2015).

37 Vu, L. P. *et al.* The N(6)-methyladenosine (m(6)A)-forming enzyme METTL3 controls myeloid differentiation of normal hematopoietic and leukemia cells. *Nat Med* **23**, 1369-1376 https://doi.org/10.1038/nm.4416 (2017).

38 Linder, B. *et al.* Single-nucleotide-resolution mapping of m6A and m6Am throughout the transcriptome. *Nature Methods* **12**, 767-772 https://doi.org/10.1038/nmeth.3453 (2015).

39 Ke, S. *et al.* m(6)A mRNA modifications are deposited in nascent pre-mRNA and are not required for splicing but do specify cytoplasmic turnover. *Genes Dev* **31**, 990-1006 https://doi.org/10.1101/gad.301036.117 (2017).

40 Shu, X. *et al.* A metabolic labeling method detects m(6)A transcriptome-wide at single base resolution. *Nature chemical biology* **16**, 887-895 https://doi.org/10.1038/s41589-020-0526-9 (2020).

41 Garcia-Campos, M. A. *et al.* Deciphering the "m(6)A Code" via Antibody-Independent Quantitative Profiling. *Cell* **178**, 731-747.e716 https://doi.org/10.1016/j.cell.2019.06.013 (2019).

42 Koh, C. W. Q., Goh, Y. T. & Goh, W. S. S. Atlas of quantitative single-base-resolution N6-methyl-adenine methylomes. *Nature Communications* **10**, 5636 https://doi.org/10.1038/s41467-019-13561-z (2019).

43 Pratanwanich, P. N. *et al.* Identification of differential RNA modifications from nanopore direct RNA sequencing with xPore. *Nature biotechnology* **39**, 1394-1402 https://doi.org/10.1038/s41587-021-00949-w (2021).

44 Liu, H. *et al.* Accurate detection of m6A RNA modifications in native RNA sequences. *Nature communications* **10**, 4079 (2019). https://doi.org/10.1038/s41467-019-11713-9

45 Parker, M. T., Barton, G. J. & Simpson, G. G. Yanocomp: robust prediction of m^6A modifications in individual nanopore direct RNA reads. Preprint at *bioRxiv* https://doi.org/10.1101/2021.06.15.448494 (2021).

46 Liu, H., Begik, O. & Novoa, E. M. EpiNano: Detection of m^6A RNA Modifications Using Oxford Nanopore Direct RNA Sequencing. *Methods in molecular biology*, 31-52 https://doi.org/10.1007/978-1-0716-1374-0_3 (2021).

47 Tang, Y. *et al.* m6A-Atlas: a comprehensive knowledgebase for unraveling the N 6-methyladenosine (m6A) epitranscriptome. *Nucleic Acids Research* **49**, D134-D143 https://doi.org/10.1093/nar/gkaa692 (2021).

48 Brawand, D. *et al.* The evolution of gene expression levels in mammalian organs. *Nature* **478**, 343-348 https://doi.org/10.1038/nature10532 (2011).

49 Barbosa-Morais, N. L. *et al.* The evolutionary landscape of alternative splicing in vertebrate species. *Science* **338**, 1587-1593 https://doi.org/10.1126/science.1230612 (2012).

50 Han, H. *et al.* MBNL proteins repress ES-cell-specific alternative splicing and reprogramming. *Nature* **498**, 241-245 https://doi.org/10.1038/nature12270 (2013).

51 Stenson, P. D. *et al.* The Human Gene Mutation Database: 2008 update. *Genome Med* **1**, 13 https://doi.org/10.1186/gm13 (2009).

52 Peltomäki, P. & Vasen, H. Mutations associated with HNPCC predisposition--Update of ICG-HNPCC/INSiGHT mutation database. *Disease markers* **20**, 269-276 https://doi.org/10.1155/2004/305058 (2004).

53 Wang, K., Li, M. & Hakonarson, H. ANNOVAR: functional annotation of genetic variants from high-throughput sequencing data. *Nucleic acids research* **38**, e164-e164 https://doi.org/10.1093/nar/gkq603 (2010).

54 Keane, T. M. *et al.* Mouse genomic variation and its effect on phenotypes and gene regulation. *Nature* **477**, 289-294 https://doi.org/10.1038/nature10413 (2011).

55 Derti, A. *et al.* A quantitative atlas of polyadenylation in five mammals. *Genome research* **22**, 1173-1183 https://doi.org/10.1101/gr.132563.111 (2012).

56 Lianoglou, S., Garg, V., Yang, J. L., Leslie, C. S. & Mayr, C. Ubiquitously transcribed genes use alternative polyadenylation to achieve tissue-specific expression. *Genes & development* **27**, 2380-2396 https://doi.org/10.1101/gad.229328.113 (2013).
57 Müller, S. *et al.* APADB: a database for alternative polyadenylation and microRNA regulation events. *Database* **2014**, bau076 https://doi.org/10.1093/database/bau076 (2014).
58 Lee, J. Y., Yeh, I., Park, J. Y. & Tian, B. PolyA_DB 2: mRNA polyadenylation sites in vertebrate genes. *Nucleic acids research* **35**, D165-D168 https://doi.org/10.1093/nar/gkl870 (2007).
59 Landrum, M. J. *et al.* ClinVar: public archive of relationships among sequence variation and human phenotype. *Nucleic Acids Res* **42**, D980-985 https://doi.org/10.1093/nar/gkt1113 (2014).
60 Loke, J. C. *et al.* Compilation of mRNA Polyadenylation Signals in Arabidopsis Revealed a New Signal Element and Potential Secondary Structures. *Plant Physiology* **138**, 1457-1468 https://doi.org/10.1104/pp.105.060541 (2005).
61 Rhee, S. Y. *et al.* The Arabidopsis Information Resource (TAIR): a model organism database providing a centralized, curated gateway to Arabidopsis biology, research materials and community. *Nucleic Acids Res* **31**, 224-228 https://doi.org/10.1093/nar/gkg076 (2003).
62 Mathelier, A. *et al.* JASPAR 2016: a major expansion and update of the open-access database of transcription factor binding profiles. *Nucleic Acids Res* **44**, D110-115 https://doi.org/10.1093/nar/gkv1176 (2016).
63 Sonnenburg, S., Schweikert, G., Philips, P., Behr, J. & Rätsch, G. Accurate splice site prediction using support vector machines. *BMC Bioinformatics* **8 Suppl 10**, S7 https://doi.org/10.1186/1471-2105-8-s10-s7 (2007).
64 Bari, A., Reaz, M. R. & Jeong, B.-S. Effective DNA encoding for splice site prediction using SVM. *MATCH Commun Math Comput Chem* **71**, 241-258 https://match.pmf.kg.ac.rs/electronic_versions/Match71/n1/match71n1_241-258.pdf (2014).
65 Degroeve, S., Saeys, Y., De Baets, B., Rouzé, P. & Van de Peer, Y. SpliceMachine: predicting splice sites from high-dimensional local context representations. *Bioinformatics* **21**, 1332-1338 https://doi.org/10.1093/bioinformatics/bti166 (2005).
66 Lee, T., & Yoon, S. Boosted categorical restricted Boltzmann machine for computational prediction of splice junctions. *Proceedings of the 32nd International Conference on Machine Learning*, PMLR **37**,2483-2492 https://proceedings.mlr.press/v37/leeb15.html (2015).
67 Mercer, T. R. *et al.* Genome-wide discovery of human splicing branchpoints. *Genome Res* **25**, 290-303 https://doi.org/10.1101/gr.182899.114 (2015).
68 Kalkatawi, M. *et al.* Dragon PolyA Spotter: predictor of poly(A) motifs within human genomic DNA sequences. *Bioinformatics* **28**, 127-129 https://doi.org/10.1093/bioinformatics/btr602 (2012).
69 Magana-Mora, A., Kalkatawi, M. & Bajic, V. B. Omni-PolyA: a method and tool for accurate recognition of Poly(A) signals in human genomic DNA. *BMC Genomics* **18**, 620 https://doi.org/10.1186/s12864-017-4033-7 (2017).
70 Temple, G. *et al.* The completion of the Mammalian Gene Collection (MGC). *Genome Res* **19**, 2324-2333 https://doi.org/10.1101/gr.095976.109 (2009).
71 Gramates, L. S. *et al.* FlyBase at 25: looking to the future. *Nucleic Acids Res* **45**, D663-D671 https://doi.org/10.1093/nar/gkw1016 (2017).
72 Leung, M. K. K., Delong, A. & Frey, B. J. Inference of the human polyadenylation code. *Bioinformatics* **34**, 2889-2898 https://doi.org/10.1093/bioinformatics/bty211 (2018).
73 Lappalainen, T. *et al.* Transcriptome and genome sequencing uncovers functional variation in humans. *Nature* **501**, 506-511 https://doi.org/10.1038/nature12531 (2013).
74 Yang, Y. *et al.* Determination of a comprehensive alternative splicing regulatory network and combinatorial regulation by key factors during the epithelial-to-mesenchymal transition.

Molecular and cellular biology **36**, 1704-1719 https://doi.org/10.1128/MCB.00019-16 (2016).
75 Lu, Z.-x. *et al.* Transcriptome-wide landscape of pre-mRNA alternative splicing associated with metastatic colonization. *Molecular Cancer Research* **13**, 305-318 https://doi.org/10.1158/1541-7786.MCR-14-0366 (2015).
76 Shen, S. *et al.* rMATS: Robust and flexible detection of differential alternative splicing from replicate RNA-Seq data. *Proceedings of the National Academy of Sciences* **111**, E5593-E5601 https://doi.org/doi:10.1073/pnas.1419161111 (2014).
77 Chong, R. *et al.* A multiplexed assay for exon recognition reveals that an unappreciated fraction of rare genetic variants cause large-effect splicing disruptions. *Molecular cell* **73**, 183-194. e188 https://doi.org/10.1016/j.molcel.2018.10.037 (2019).
78 McRae, J. F. *et al.* Prevalence and architecture of de novo mutations in developmental disorders. *Nature* **542**, 433-438 https://doi.org/10.1038/nature21062 (2017).
79 Fischbach, G. D. & Lord, C. The Simons Simplex Collection: A Resource for Identification of Autism Genetic Risk Factors. *Neuron* **68**, 192-195 https://doi.org/https://doi.org/10.1016/j.neuron.2010.10.006 (2010).
80 De Rubeis, S. *et al.* Synaptic, transcriptional and chromatin genes disrupted in autism. *Nature* **515**, 209-215 https://doi.org/10.1038/nature13772 (2014).
81 Rosenberg, Alexander B., Patwardhan, Rupali P., Shendure, J. & Seelig, G. Learning the Sequence Determinants of Alternative Splicing from Millions of Random Sequences. *Cell* **163**, 698-711 https://doi.org/10.1016/j.cell.2015.09.054 (2015).
82 Adamson, S. I., Zhan, L. & Graveley, B. R. Vex-seq: high-throughput identification of the impact of genetic variation on pre-mRNA splicing efficiency. *Genome Biol* **19**, 71 https://doi.org/10.1186/s13059-018-1437-x (2018).
83 Soemedi, R. *et al.* Pathogenic variants that alter protein code often disrupt splicing. *Nature Genetics* **49**, 848-855 https://doi.org/10.1038/ng.3837 (2017).
84 Kalkatawi, M., Magana-Mora, A., Jankovic, B. & Bajic, V. B. DeepGSR: an optimized deep-learning structure for the recognition of genomic signals and regions. *Bioinformatics* **35**, 1125-1132 https://doi.org/10.1093/bioinformatics/bty752 (2019).
85 Xia, Z. *et al.* DeeReCT-PolyA: a robust and generic deep learning method for PAS identification. *Bioinformatics* **35**, 2371-2379 https://doi.org/10.1093/bioinformatics/bty991 (2019).
86 Palomares, M.-A. *et al.* Systematic analysis of TruSeq, SMARTer and SMARTer Ultra-Low RNA-seq kits for standard, low and ultra-low quantity samples. *Scientific Reports* **9**, 7550 https://doi.org/10.1038/s41598-019-43983-0 (2019).
87 Schuierer, S. *et al.* A comprehensive assessment of RNA-seq protocols for degraded and low-quantity samples. *BMC Genomics* **18**, 442 https://doi.org/10.1186/s12864-017-3827-y (2017).
88 Saba, L. M. *et al.* The sequenced rat brain transcriptome--its use in identifying networks predisposing alcohol consumption. *The FEBS journal* **282**, 3556-3578 https://doi.org/10.1111/febs.13358 (2015).
89 Masamha, C. P. *et al.* CFIm25 links alternative polyadenylation to glioblastoma tumour suppression. *Nature* **510**, 412-416 https://doi.org/10.1038/nature13261 (2014).
90 Li, B. *et al.* A comprehensive mouse transcriptomic BodyMap across 17 tissues by RNA-seq. *Scientific reports* **7**, 4200 https://doi.org/10.1038/s41598-017-04520-z (2017).
91 Lawrence, C. J., Dong, Q., Polacco, M. L., Seigfried, T. E. & Brendel, V. MaizeGDB, the community database for maize genetics and genomics. *Nucleic Acids Res* **32**, D393-397 https://doi.org/10.1093/nar/gkh011 (2004).
92 Cardoso-Moreira, M. *et al.* Gene expression across mammalian organ development. *Nature* **571**, 505-509 https://doi.org/10.1038/s41586-019-1338-5 (2019).

93 MacArthur, J. *et al.* The new NHGRI-EBI Catalog of published genome-wide association studies (GWAS Catalog). *Nucleic Acids Res* **45**, D896-D901 https://doi.org/10.1093/nar/gkw1133 (2017).
94 Cherry, J. M. *et al.* SGD: Saccharomyces Genome Database. *Nucleic Acids Res* **26**, 73-79 https://doi.org/10.1093/nar/26.1.73 (1998).
95 Cherry, J. M. *et al.* Saccharomyces Genome Database: the genomics resource of budding yeast. *Nucleic Acids Res* **40**, D700-705 https://doi.org/10.1093/nar/gkr1029 (2012).
96 Xu, Z. *et al.* Bidirectional promoters generate pervasive transcription in yeast. *Nature* **457**, 1033-1037 https://doi.org/10.1038/nature07728 (2009).
97 Zrimec, J. *et al.* Deep learning suggests that gene expression is encoded in all parts of a co-evolving interacting gene regulatory structure. *Nature Communications* **11**, 6141 https://doi.org/10.1038/s41467-020-19921-4 (2020).
98 Santos-Zavaleta, A. *et al.* RegulonDB v 10.5: tackling challenges to unify classic and high throughput knowledge of gene regulation in E. coli K-12. *Nucleic Acids Res* **47**, D212-d220 https://doi.org/10.1093/nar/gky1077 (2019).
99 Ziemann, M., Kaspi, A. & El-Osta, A. Digital expression explorer 2: a repository of uniformly processed RNA sequencing data. *GigaScience* **8** https://doi.org/10.1093/gigascience/giz022 (2019).
100 Keren, L. *et al.* Promoters maintain their relative activity levels under different growth conditions. *Mol Syst Biol* **9**, 701 https://doi.org/10.1038/msb.2013.59 (2013).
101 Yamanishi, M. *et al.* A genome-wide activity assessment of terminator regions in Saccharomyces cerevisiae provides a ''terminatome'' toolbox. *ACS Synth Biol* **2**, 337-347 https://doi.org/10.1021/sb300116y (2013).
102 Khan, A. *et al.* JASPAR 2018: update of the open-access database of transcription factor binding profiles and its web framework. *Nucleic Acids Res* **46**, D260-D266 https://doi.org/10.1093/nar/gkx1126 (2018).
103 Teixeira, M. C. *et al.* YEASTRACT: an upgraded database for the analysis of transcription regulatory networks in Saccharomyces cerevisiae. *Nucleic Acids Res* **46**, D348-D353 https://doi.org/10.1093/nar/gkx842 (2018).
104 Yevshin, I., Sharipov, R., Valeev, T., Kel, A. & Kolpakov, F. GTRD: a database of transcription factor binding sites identified by ChIP-seq experiments. *Nucleic Acids Res* **45**, D61-D67 https://doi.org/10.1093/nar/gkw951 (2017).
105 Agarwal, V., Bell, G. W., Nam, J. W. & Bartel, D. P. Predicting effective microRNA target sites in mammalian mRNAs. *Elife* **4** https://doi.org/10.7554/eLife.05005 (2015).
106 Lek, M. *et al.* Analysis of protein-coding genetic variation in 60,706 humans. *Nature* **536**, 285-291 https://doi.org/10.1038/nature19057 (2016).
107 Smith, C. L. *et al.* Mouse Genome Database (MGD)-2018: knowledgebase for the laboratory mouse. *Nucleic Acids Res* **46**, D836-D842 https://doi.org/10.1093/nar/gkx1006 (2018).
108 Koscielny, G. *et al.* The International Mouse Phenotyping Consortium Web Portal, a unified point of access for knockout mice and related phenotyping data. *Nucleic Acids Res* **42**, D802-809 https://doi.org/10.1093/nar/gkt977 (2014).
109 Tsherniak, A. *et al.* Defining a Cancer Dependency Map. *Cell* **170**, 564-576 e516 https://doi.org/10.1016/j.cell.2017.06.010 (2017).
110 Liberzon, A. *et al.* The Molecular Signatures Database (MSigDB) hallmark gene set collection. *Cell Syst* **1**, 417-425 https://doi.org/10.1016/j.cels.2015.12.004 (2015).
111 Cusanovich, D. A. *et al.* A Single-Cell Atlas of In Vivo Mammalian Chromatin Accessibility. *Cell* **174**, 1309-1324 e1318 https://doi.org/10.1016/j.cell.2018.06.052 (2018).
112 Kim, S. S. *et al.* Improving the informativeness of Mendelian disease-derived pathogenicity scores for common disease. *Nat Commun* **11**, 6258 https://doi.org/10.1038/s41467-020-20087-2 (2020).

113 Wang, G., Sarkar, A., Carbonetto, P. & Stephens, M. A simple new approach to variable selection in regression, with application to genetic fine mapping. *J R Stat Soc Series B Stat Methodol* **82**, 1273-1300 https://doi.org/10.1111/rssb.12388 (2020).
114 Schofield, J. A., Duffy, E. E., Kiefer, L., Sullivan, M. C. & Simon, M. D. TimeLapse-seq: adding a temporal dimension to RNA sequencing through nucleoside recoding. *Nat Methods* **15**, 221-225 https://doi.org/10.1038/nmeth.4582 (2018).
115 Herzog, V. A. *et al.* Thiol-linked alkylation of RNA to assess expression dynamics. *Nat Methods* **14**, 1198-1204 https://doi.org/10.1038/nmeth.4435 (2017).
116 Denzler, R. *et al.* Impact of MicroRNA Levels, Target-Site Complementarity, and Cooperativity on Competing Endogenous RNA-Regulated Gene Expression. *Mol Cell* **64**, 565-579 https://doi.org/10.1016/j.molcel.2016.09.027 (2016).
117 van Arensbergen, J. *et al.* Genome-wide mapping of autonomous promoter activity in human cells. *Nat Biotechnol* **35**, 145-153 https://doi.org/10.1038/nbt.3754 (2017).
118 Kelley, D. R. Cross-species regulatory sequence activity prediction. *PLOS Computational Biology* **16**, e1008050 https://doi.org/10.1371/journal.pcbi.1008050 (2020).
119 Shigaki, D. *et al.* Integration of multiple epigenomic marks improves prediction of variant impact in saturation mutagenesis reporter assay. *Hum Mutat* **40**, 1280-1291 https://doi.org/10.1002/humu.23797 (2019).
120 Fulco, C. P. *et al.* Activity-by-contact model of enhancer-promoter regulation from thousands of CRISPR perturbations. *Nat Genet* **51**, 1664-1669 https://doi.org/10.1038/s41588-019-0538-0 (2019).
121 Gasperini, M., Tome, J. M. & Shendure, J. Towards a comprehensive catalogue of validated and target-linked human enhancers. *Nat Rev Genet* **21**, 292-310 https://doi.org/10.1038/s41576-019-0209-0 (2020).
122 Fudenberg, G., Kelley, D. R. & Pollard, K. S. Predicting 3D genome folding from DNA sequence with Akita. *Nat Methods* **17**, 1111-1117 https://doi.org/10.1038/s41592-020-0958-x (2020).
123 Wang, Q. S. *et al.* Leveraging supervised learning for functionally informed fine-mapping of cis-eQTLs identifies an additional 20,913 putative causal eQTLs. *Nat Commun* **12**, 3394 https://doi.org/10.1038/s41467-021-23134-8 (2021).
124 Fadista, J. *et al.* Global genomic and transcriptomic analysis of human pancreatic islets reveals novel genes influencing glucose metabolism. *Proc Natl Acad Sci U S A* **111**, 13924-13929 https://doi.org/10.1073/pnas.1402665111 (2014).
125 Replogle, J. M. *et al.* Mapping information-rich genotype-phenotype landscapes with genome-scale Perturb-seq. *Cell* **185**, 2559-2575 e2528 https://doi.org/10.1016/j.cell.2022.05.013 (2022).
126 Vaishnav, E. D. *et al.* The evolution, evolvability and engineering of gene regulatory DNA. *Nature* **603**, 455-463 https://doi.org/10.1038/s41586-022-04506-6 (2022).
127 Tothill, R. W. *et al.* Novel molecular subtypes of serous and endometrioid ovarian cancer linked to clinical outcome. *Clin Cancer Res* **14**, 5198-5208 https://doi.org/10.1158/1078-0432.CCR-08-0196 (2008).
128 Curtis, C. *et al.* The genomic and transcriptomic architecture of 2,000 breast tumours reveals novel subgroups. *Nature* **486**, 346-352 https://doi.org/10.1038/nature10983 (2012).
129 Kather, J. N. *et al.* Predicting survival from colorectal cancer histology slides using deep learning: A retrospective multicenter study. *PLoS Med* **16**, e1002730 https://doi.org/10.1371/journal.pmed.1002730 (2019).
130 Saillard, C. *et al.* Predicting Survival After Hepatocellular Carcinoma Resection Using Deep Learning on Histological Slides. *Hepatology* **72**, 2000-2013 https://doi.org/10.1002/hep.31207 (2020).

131 Bulten, W. *et al.* Epithelium segmentation using deep learning in H&E-stained prostate specimens with immunohistochemistry as reference standard. *Sci Rep* **9**, 864 https://doi.org/10.1038/s41598-018-37257-4 (2019).
132 Bakr, S. *et al.* A radiogenomic dataset of non-small cell lung cancer. *Sci Data* **5**, 180202 https://doi.org/10.1038/sdata.2018.202 (2018).
133 Croft, D. *et al.* The Reactome pathway knowledgebase. *Nucleic Acids Res* **42**, D472-477 https://doi.org/10.1093/nar/gkt1102 (2014).
134 Slenter, D. N. *et al.* WikiPathways: a multifaceted pathway database bridging metabolomics to other omics research. *Nucleic Acids Res* **46**, D661-D667 https://doi.org/10.1093/nar/gkx1064 (2018).
135 Kim, H. K. *et al.* SpCas9 activity prediction by DeepSpCas9, a deep learning-based model with high generalization performance. *Sci Adv* **5**, eaax9249 https://doi.org/10.1126/sciadv.aax9249 (2019).
136 Bennett, D. A., Schneider, J. A., Arvanitakis, Z. & Wilson, R. S. Overview and findings from the religious orders study. *Curr Alzheimer Res* **9**, 628-645 https://doi.org/10.2174/156720512801322573 (2012).
137 De Jager, P. L. *et al.* A multi-omic atlas of the human frontal cortex for aging and Alzheimer's disease research. *Sci Data* **5**, 180142 https://doi.org/10.1038/sdata.2018.142 (2018).
138 Parker, J. S. *et al.* Supervised risk predictor of breast cancer based on intrinsic subtypes. *J Clin Oncol* **27**, 1160-1167 https://doi.org/10.1200/JCO.2008.18.1370 (2009).
139 Cancer Genome Atlas, N. Comprehensive molecular portraits of human breast tumours. *Nature* **490**, 61-70 https://doi.org/10.1038/nature11412 (2012).
140 Song, M. *et al.* Sequence-specific prediction of the efficiencies of adenine and cytosine base editors. *Nat Biotechnol* **38**, 1037-1043 https://doi.org/10.1038/s41587-020-0573-5 (2020).
141 Arbab, M. *et al.* Determinants of Base Editing Outcomes from Target Library Analysis and Machine Learning. *Cell* **182**, 463-480 e430 https://doi.org/10.1016/j.cell.2020.05.037 (2020).
142 Leppek, K. *et al.* Combinatorial optimization of mRNA structure, stability, and translation for RNA-based therapeutics. *Nat Commun* **13**, 1536 https://doi.org/10.1038/s41467-022-28776-w (2022).
143 Yang, W. *et al.* Genomics of Drug Sensitivity in Cancer (GDSC): a resource for therapeutic biomarker discovery in cancer cells. *Nucleic Acids Res* **41**, D955-961 https://doi.org/10.1093/nar/gks1111 (2013).
144 Iorio, F. *et al.* A Landscape of Pharmacogenomic Interactions in Cancer. *Cell* **166**, 740-754 https://doi.org/10.1016/j.cell.2016.06.017 (2016).
145 Wessels, H. H. *et al.* Massively parallel Cas13 screens reveal principles for guide RNA design. *Nat Biotechnol* **38**, 722-727 https://doi.org/10.1038/s41587-020-0456-9 (2020).
146 Anzalone, A. V. *et al.* Search-and-replace genome editing without double-strand breaks or donor DNA. *Nature* **576**, 149-157 https://doi.org/10.1038/s41586-019-1711-4 (2019).
147 Kim, H. K. *et al.* Predicting the efficiency of prime editing guide RNAs in human cells. *Nat Biotechnol* **39**, 198-206 https://doi.org/10.1038/s41587-020-0677-y (2021).
148 Li, Y., Chen, J., Tsai, S. Q. & Cheng, Y. Easy-Prime: a machine learning-based prime editor design tool. *Genome Biol* **22**, 235 https://doi.org/10.1186/s13059-021-02458-0 (2021).
149 He, W. *et al.* Accelerating bioactive peptide discovery via mutual information-based meta-learning. *Brief Bioinform* **23** https://doi.org/10.1093/bib/bbab499 (2022).
150 Luo, X. *et al.* SPENCER: a comprehensive database for small peptides encoded by noncoding RNAs in cancer patients. *Nucleic Acids Res* **50**, D1373-D1381 https://doi.org/10.1093/nar/gkab822 (2022).
151 Vaske, O. M. *et al.* Comparative Tumor RNA Sequencing Analysis for Difficult-to-Treat Pediatric and Young Adult Patients With Cancer. *JAMA Netw Open* **2**, e1913968 https://doi.org/10.1001/jamanetworkopen.2019.13968 (2019).

152 McLeod, C. *et al.* St. Jude Cloud: A Pediatric Cancer Genomic Data-Sharing Ecosystem. *Cancer Discov* **11**, 1082-1099 https://doi.org/10.1158/2159-8290.CD-20-1230 (2021).
153 Auyeung, V. C., Ulitsky, I., McGeary, S. E. & Bartel, D. P. Beyond secondary structure: primary-sequence determinants license pri-miRNA hairpins for processing. *Cell* **152**, 844-858 https://doi.org/10.1016/j.cell.2013.01.031 (2013).
154 Danaee, P. *et al.* bpRNA: large-scale automated annotation and analysis of RNA secondary structure. *Nucleic acids research* **46**, 5381-5394 https://doi.org/10.1093/nar/gky285 (2018).
155 Schaum, N. *et al.* Single-cell transcriptomics of 20 mouse organs creates a Tabula Muris: The Tabula Muris Consortium. *Nature* **562**, 367 https://doi.org/10.1038/s41586-018-0590-4 (2018).
156 Almanzar, N. *et al.* A single-cell transcriptomic atlas characterizes ageing tissues in the mouse. *Nature* **583**, 590-595 https://doi.org/10.1038/s41586-020-2496-1 (2020).
157 Das, R. & Baker, D. Automated de novo prediction of native-like RNA tertiary structures. *Proceedings of the National Academy of Sciences* **104**, 14664-14669 https://doi.org/doi:10.1073/pnas.0703836104 (2007).
158 Watkins, A. M., Rangan, R. & Das, R. FARFAR2: Improved De Novo Rosetta Prediction of Complex Global RNA Folds. *Structure* **28**, 963-976.e966 https://doi.org/https://doi.org/10.1016/j.str.2020.05.011 (2020).
159 Cruz, J. A. *et al.* RNA-Puzzles: a CASP-like evaluation of RNA three-dimensional structure prediction. *RNA* **18**, 610-625 https://doi.org/10.1261/rna.031054.111 (2012).
160 Kalvari, I. *et al.* Rfam 14: expanded coverage of metagenomic, viral and microRNA families. *Nucleic Acids Research* **49**, D192-D200 https://doi.org/10.1093/nar/gkaa1047 (2021).
161 Ma, S. *et al.* Caloric Restriction Reprograms the Single-Cell Transcriptional Landscape of Rattus Norvegicus Aging. *Cell* **180**, 984-1001.e1022 https://doi.org/10.1016/j.cell.2020.02.008 (2020).
162 Dreos, R., Ambrosini, G., Cavin Périer, R. & Bucher, P. EPD and EPDnew, high-quality promoter resources in the next-generation sequencing era. *Nucleic Acids Res* **41**, D157-164 https://doi.org/10.1093/nar/gks1233 (2013).
163 Yoon, H. *et al.* Gene expression profiling of isogenic cells with different TP53 gene dosage reveals numerous genes that are affected by TP53 dosage and identifies CSPG2 as a direct target of p53. *Proceedings of the National Academy of Sciences* **99**, 15632-15637 https://doi.org/doi:10.1073/pnas.242597299 (2002).
164 Koeppel, M. *et al.* Crosstalk between c-Jun and TAp73alpha/beta contributes to the apoptosis-survival balance. *Nucleic Acids Res* **39**, 6069-6085 https://doi.org/10.1093/nar/gkr028 (2011).
165 Wang, R., Wang, Z., Wang, J. & Li, S. SpliceFinder: ab initio prediction of splice sites using convolutional neural network. *BMC Bioinformatics* **20**, 652 https://doi.org/10.1186/s12859-019-3306-3 (2019).
166 Franzén, O., Gan, L.-M. & Björkegren, J. L. PanglaoDB: a web server for exploration of mouse and human single-cell RNA sequencing data. *Database* **2019**, baz046 https://doi.org/10.1093/database/baz046 (2019).
167 Litviňuková, M. *et al.* Cells of the adult human heart. *Nature* **588**, 466-472 https://doi.org/10.1038/s41586-020-2797-4 (2020).
168 Zheng, G. X. *et al.* Massively parallel digital transcriptional profiling of single cells. *Nature communications* **8**, 14049 https://doi.org/10.1038/ncomms14049 (2017).
169 Zheng, R. *et al.* Cistrome Data Browser: expanded datasets and new tools for gene regulatory analysis. *Nucleic Acids Research* **47**, D729-D735 https://doi.org/10.1093/nar/gky1094 (2019).
170 Hibbs, M. A. *et al.* Exploring the functional landscape of gene expression: directed search of large microarray compendia. *Bioinformatics* **23**, 2692-2699 https://doi.org/10.1093/bioinformatics/btm403 (2007).

171 Costanzo, M. *et al.* A global genetic interaction network maps a wiring diagram of cellular function. *Science* **353**, aaf1420 https://doi.org/10.1126/science.aaf1420 (2016).
172 Orchard, S. *et al.* The MIntAct project--IntAct as a common curation platform for 11 molecular interaction databases. *Nucleic Acids Res* **42**, D358-363 https://doi.org/10.1093/nar/gkt1115 (2014).
173 Piotrowski, J. S. *et al.* Functional annotation of chemical libraries across diverse biological processes. *Nature chemical biology* **13**, 982-993 https://doi.org/10.1038/nchembio.2436 (2017).
174 Sweeney, B. A. *et al.* RNAcentral 2021: secondary structure integration, improved sequence search and new member databases. *Nucleic Acids Research* 49, D212-D220 https://doi.org/10.1093/nar/gkaa921 (2020).
175 Sloma, M. F. & Mathews, D. H. Exact calculation of loop formation probability identifies folding motifs in RNA secondary structures. *RNA* **22**, 1808-1818 https://doi.org/10.1261/rna.053694.115 (2016).
176 Tan, Z., Fu, Y., Sharma, G. & Mathews, D. H. TurboFold II: RNA structural alignment and secondary structure prediction informed by multiple homologs. *Nucleic Acids Res* **45**, 11570-11581 https://doi.org/10.1093/nar/gkx815 (2017).
177 Sun, S., Wang, W., Peng, Z. & Yang, J. RNA inter-nucleotide 3D closeness prediction by deep residual neural networks. *Bioinformatics* **37**, 1093-1098 https://doi.org/10.1093/bioinformatics/btaa932 (2021).
178 Sample, P. J. *et al.* Human 5′ UTR design and variant effect prediction from a massively parallel translation assay. *Nature Biotechnology* **37**, 803-809 https://doi.org/10.1038/s41587-019-0164-5 (2019).
179 Auton, A. *et al.* A global reference for human genetic variation. *Nature* **526**, 68-74 https://doi.org/10.1038/nature15393 (2015).
180 Collins, R. L. *et al.* A cross-disorder dosage sensitivity map of the human genome. *Cell* **185**, 3041-3055. e3025 https://doi.org/10.1016/j.cell.2022.06.036 (2022).
181 Pan, G. *et al.* Whole-Genome Analysis of Histone H3 Lysine 4 and Lysine 27 Methylation in Human Embryonic Stem Cells. *Cell Stem Cell* **1**, 299-312 https://doi.org/https://doi.org/10.1016/j.stem.2007.08.003 (2007).
182 Herrmann, C. J. *et al.* PolyASite 2.0: a consolidated atlas of polyadenylation sites from 3' end sequencing. *Nucleic Acids Res* **48**, D174-d179 https://doi.org/10.1093/nar/gkz918 (2020).
183 Li, K. *et al.* Performance evaluation of differential splicing analysis methods and splicing analytics platform construction. *Nucleic Acids Research* **50**, 9115-9126 https://doi.org/10.1093/nar/gkac686 (2022).
184 Swanson, E. *et al.* Simultaneous trimodal single-cell measurement of transcripts, epitopes, and chromatin accessibility using TEA-seq. *eLife* **10**, e63632 https://doi.org/10.7554/eLife.63632 (2021).
185 Mimitou, E. P. *et al.* Scalable, multimodal profiling of chromatin accessibility, gene expression and protein levels in single cells. *Nat Biotechnol* **39**, 1246-1258 https://doi.org/10.1038/s41587-021-00927-2 (2021).
186 Ding, J. *et al.* Systematic comparison of single-cell and single-nucleus RNA-sequencing methods. *Nat Biotechnol* **38**, 737-746 https://doi.org/10.1038/s41587-020-0465-8 (2020).
187 Satpathy, A. T. *et al.* Massively parallel single-cell chromatin landscapes of human immune cell development and intratumoral T cell exhaustion. *Nat Biotechnol* **37**, 925-936 https://doi.org/10.1038/s41587-019-0206-z (2019).
